# Knowledge mapping and current trends of global research on CRISPR in the field of cancer

**DOI:** 10.3389/fcell.2023.1178221

**Published:** 2023-05-02

**Authors:** Han Liu, Zongwei Lv, Gong Zhang, Xia Wang, Yuan Wang, Kefeng Wang

**Affiliations:** ^1^ Department of Urology, Shengjing Hospital of China Medical University, Shenyang, China; ^2^ Department of General Surgery, Shengjing Hospital of China Medical University, Shenyang, China

**Keywords:** bibliometrics, CRISPR, cancer, CAS, immunotherapy

## Abstract

**Background:** Gene editing tools using clustered regularly interspaced short palindromic repeats (CRISPR) and CRISPR-related systems have revolutionized our understanding of cancer. The purpose of this study was to determine the distribution, collaboration, and direction of cancer research using CRISPR.

**Methods:** Data from the Web of Science (WoS) Core Collection database were collected from 4,408 cancer publications related to CRISPR from 1 January 2013to 31 December 2022. The obtained data were analyzed using VOSviewer software for citation, co-citation, co-authorship, and co-occurrence analysis.

**Results:** The number of annual publications has grown steadily over the past decade worldwide. The United States was shown, by far, to be the leading source of cancer publications, citations, and collaborations involving CRISPR than any other country, followed by China. Li Wei (Jilin University, China), and Harvard Medical School (Boston, MA, United States) were the author and institution with the most publications and active collaborations, respectively. The journal with the most contributions was *Nature Communications* (*n* = 147) and the journal with the most citations was *Nature* (*n* = 12,111). The research direction of oncogenic molecules, mechanisms, and cancer-related gene editing was indicated based on keyword analysis.

**Conclusion:** The current study has provided a comprehensive overview of cancer research highlights and future trends of CRISPR, combined with a review of CRISPR applications in cancer to summarize and predict research directions and provide guidance to researchers.

## 1 Introduction

Cancer is a complicated and hereditary disease that has attracted worldwide attention. Studies have shown that tumorigenesis is comprised of cumulative somatic mutations and epigenetic aberrations of oncogenes and tumor suppressor genes (Garraway and Lander, 2013; Sanjana et al., 2014). Cancer kills one in six people worldwide and threatens thousands of lives (Stupp et al., 2009; Vogelstein et al., 2013; Sung et al., 2021). With the discovery of high-throughput sequencing technology, a large number of genes related to the occurrence and development of cancer have been discovered over the past 20 years (Pon and Marra, 2015). Gene editing technology has facilitated these advances in cancer research (Drost et al., 2017) and further helped to identify therapeutic targets (Manguso et al., 2017).

Several techniques have been applied to achieve gene editing. Since the discovery of RNA programmability and mammalian cell adaptation (Jinek et al., 2012; Cong et al., 2013), clustered regularly interspaced short palindromic repeats (CRISPR) technology and CRISPR-associated system (Cas) have come to be recognized as a revolutionary gene editing toolkit (Zhang et al., 2021). The CRISPR-Cas is an RNA-mediated adaptive immune system that provides acquired immunity against invading viruses and phages in bacteria and archaea (Barrangou et al., 2007; Garneau et al., 2010). Because of the advantages of a simple and rapid design, the CRISPR-Cas has been widely used, especially in cancer biology research (Katti et al., 2022). Gene editing tools have had a major impact on cancer biology and are emerging as a promising approach to cancer diagnosis and treatment.

Given the rapid development of CRISPR in cancer research, various detailed reviews are emerging. Although some conclusions are clear and insightful, there is a lack of complete and macro-quantitative research. In contrast, bibliometrics is a new statistical and mathematical approach used to analyze scientific outputs, thus providing investigators with both qualitative and quantitative characteristics (Wallin, 2005; Donthu et al., 2021). Bibliometrics allows large-scale, objective summaries of existing literature in an area of research, thus enabling researchers to clarify current research trends in core areas (Wallin, 2005; Chen et al., 2018).

Therefore, we conducted a systematic investigation of CRISPR-related scientific achievements in cancer from 2013 to 2022 to identify current research trends in cancer biology. Then, various bibliometrics and visual indicators were used to reveal the relevant contributions, influence, and co-authorship structure. The current study summarizes the development of CRISPR in basic cancer research and the clinical application.

## 2 Materials and methods

### 2.1 Data source and collection

**FIGURE 1:**
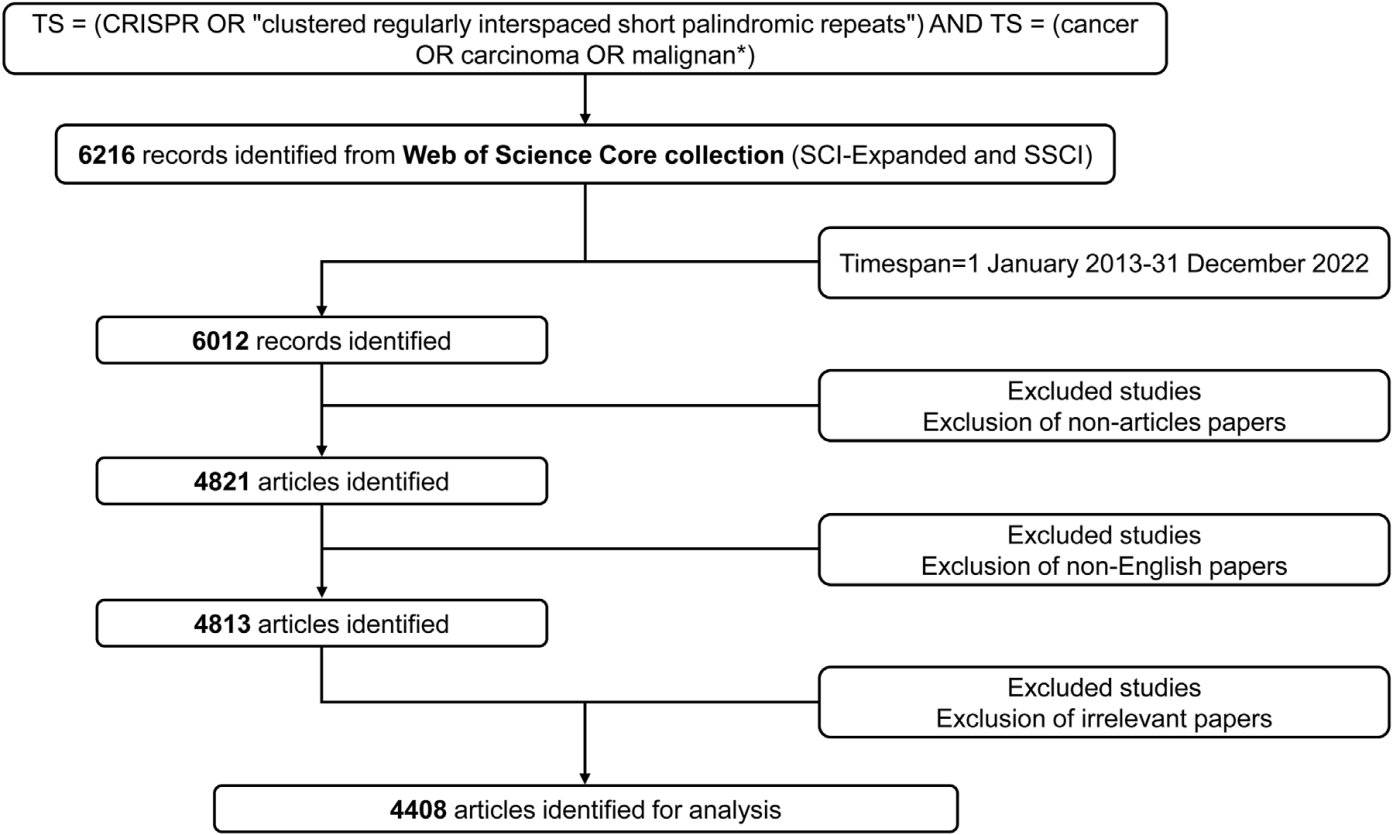
Flow diagram of literature screening related to CRISPR in cancer.

We retrieved the world’s publications that utilized CRISPR in cancer research from the Web of Science (WoS) Core Collection database (Clarivate). The detailed data retrieval and exclusion process of this study are provided in Figure 1. The search strategies in the advanced section were as follows: TS = (CRISPR OR “clustered regularly interspaced short palindromic repeats”) AND TS = (cancer OR carcinoma OR malignant*). The data spanned from 1 January 2013 to 31 December 2022. The type of publication was limited to “article” only published in English. Then, we excluded irrelevant papers and finally obtained a sample of 4,408 articles for analysis. The relevant records were exported to VOSviewer (version 1.6.18; Leiden University) as a plain text file in “full record and cited references” format.

### 2.2 Data analysis and visualization

The VOSviewer software was selected as the primary tool for comprehensive analysis and network construction to visualize and inspect large bibliometric networks using graphical presentations (van Eck and Waltman, 2010). We created visualization maps to analyze the intellectual interactions and structural connections among research constituents through different bibliometric functions. The analysis results are presented in network, overlay, and density visualization consisting of items/clusters in different colors and lines between them. The size of circles was positively correlated with the contributions of research constituents. The cluster color indicated the order of the cluster number. In addition, the circle color shade in the overlay map reflected the proximity of the year to the article. The lines between the items demonstrated the relationship and strength of the cooperation.

The WoS “analyze results” and “citation report” functions were used to perform basic publication and citation statistics, including publications over time, the times cited, and the average citations per article. The impact factor (IF) and quartile ranking were collected from the 2021 edition of *Journal Citation Reports (JCR)*. Microsoft Excel 2019 was used to draw and analyze the publications, citations trends, and major constituent (authors, journals, and countries/regions) distribution. Microsoft PowerPoint 2019 was used to draw the flowchart depicting literature screening.

## 3 Results

### 3.1 Overview of annual publications and citations

**FIGURE 2:**
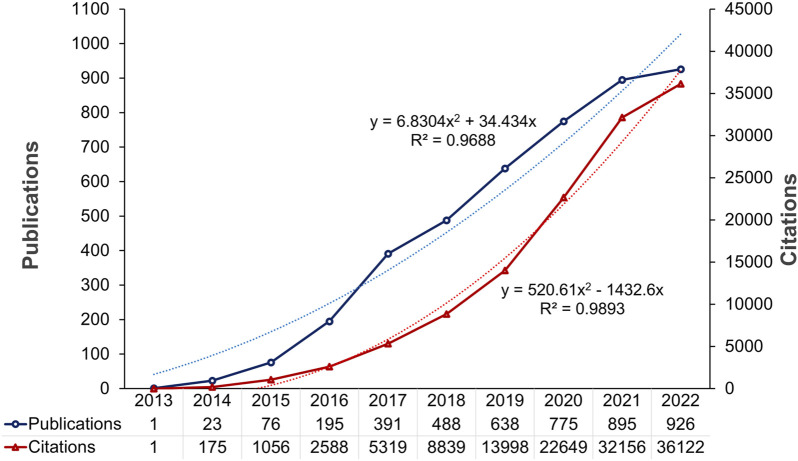
Annual trends in publications and citations on CRISPR in cancer from 2013 to 2022.

The number of publications and citations per year or per research constituent were the most prominent measures of performance analysis, representing productivity and impact, respectively (Donthu et al., 2021). Figure 2 depicts the annual trend of publications, the distribution of citations, and the function-fitting curves. From 2013 to 2022, the number of cancer-related publications involving CRISPR increased from 1 to 4,408. The increase in the number of articles was more pronounced in 2016-2017 than other years, while a slight downward trend occurred in 2021-2022. The growing trend indicated that CRISPR use in cancer research has attracted significant attention over the past 11 years.

In addition, a total of 4,408 articles were cited 128,834 times, with an average of 29.23 citations per article. Both the exponential growth in citation number and the curve-fitting upward trend demonstrated the global interest and cancer research potential in using CRISPR. In summary, we deduced that CRISPR would receive more attention and have greater cancer research prospects in the future.

### 3.2 Authors analysis

#### 3.2.1 Leading authors

**FIGURE 3:**
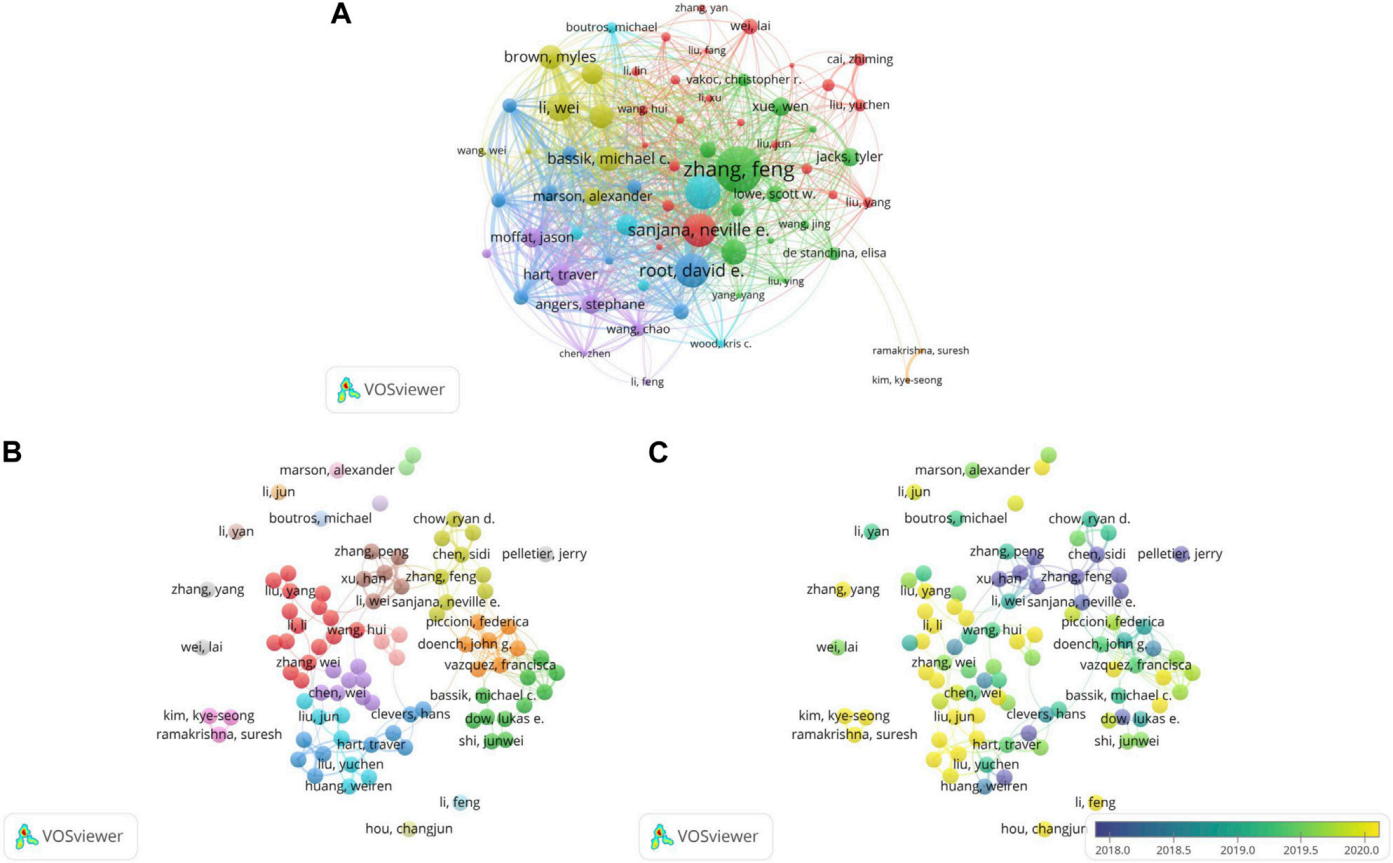
Bibliometric analysis of leading authors and co-authorships in the field of CRISPR in cancer. **(A)** The citation analysis of leading authors. **(B)** Network visualization map of the leading authors collaboration. **(C)** Overlay visualization map of the leading authors collaboration.

Greater than 30,000 researchers have contributed to CRISPR-related cancer research. Table 1 lists the top 10 most productive authors, along with the citations and citation-to-publication (C:P) ratio. Figure 3A shows a network map of author citation analysis, with node size proportional to author citation counts. Specifically, Sanjana Neville E. ranked fourth in citation number (*n* = 4,760) with relatively few articles (*n* = 10). Although the number of articles published by the author was relatively small, the high number of citations indicated that the articles were of high quality and widely recognized by the profession. As shown in Table 1, Li Wei (*n* = 36) ranked first among the top 10 active authors, followed by Hart Traver (*n* = 22) and Doench, John G. (*n* = 21), who was also the second most cited author (n = 5,228).

**TABLE 1:** The top 10 most productive authors related to CRISPR in cancer.

Rank	Author	Publications	Citations	C: P ratio
1	Li, Wei	36	2,960	82.22
2	Hart, Traver	22	1,926	87.55
3	Doench, John G	21	5,228	248.95
4	Zhang, Wei	21	484	23.05
5	Root, David E	20	4,785	239.25
6	Li, Li	19	353	18.58
7	Zhang, Feng	18	9,334	518.56
8	Chen, Sidi	17	2,708	159.29
9	Liu, Yang	17	490	28.82
10	Bassik, Michael C	16	2,508	156.75

We also determined that Zhang Feng was the most cited author (*n* = 9,334). Eighteen publications by him had a C:P ratio of 518.56, which ranked first. In addition, according to our search, Zhang Feng was the corresponding author of the two most cited papers. The most cited paper (*n* = 3,045) was an article published by Shalem, Ophir in *Science* in 2014 entitled “Genome-Scale CRISPR-Cas9 Knockout Screening in Human Cells”. Shalem et al. identified the possibility of negative/positive selection screening in human cells through lentivirus delivery of a genome scale CRISPR-Cas9 knockout library (Shalem et al., 2014). This work provided a more effective and promising method for targeted screening.

Of note, Table 1 also shows that Li Wei, the most productive author, had a C:P ratio of 82.22, which was lower than some authors. Li had the lowest citation number (*n* = 353) and C:P ratio (*n* = 18.58) among the most productive authors.

#### 3.2.2 Collaboration of authors

Figure 3B displays a network visualization map of the co-authorship analysis of 91 authors with at least 9 published articles. Among the cancer studies that utilized CRISPR, there were individual researchers in addition to groups of a few academics or many researchers.

Figure 3B depicts numerous individual researchers. Among the researchers, Li Feng and Li Yan were the most productive individual authors with 12 publications each, which was more than all other individual researchers and even some small collaborative groups. The most influential individual researcher was Marson Alexander, who had published 10 papers with 1,411 citations. Another individual researcher with a high citation number was Wei Lai (*n* = 1,046), who discovered a key driver of sorafenib resistance in hepatocellular carcinoma treatment through genome-wide CRISPR-Cas9 library screening (Wei et al., 2019). The study also indicated that resistance is effectively overcome by targeting the key driver.

The center of the illustration displays several large research groups containing most of the researchers and their collaborations. Most of the collaborators groups had primary authors, such as Zhang Feng in the yellow cluster, Li Wei in the brown cluster, Doench John G. in the orange cluster and Hart Traver in the blue cluster. By comparison, the orange cluster had the highest total link strength and the co-authors had published numerous influential articles. For example, these researchers discovered that targeting genomic-amplified regions in cancer cell lines using CRISPR-Cas9 technology induce DNA damage and G2 cell cycle arrest (Aguirre et al., 2016). This gene-independent anti-proliferative cell response may allow sequence-specific therapeutic strategies to be used in cancer therapy.

The overlay visualization map is shown in Figure 3C. The research group, consisting of Chen Sidi and Zhang Feng, started relevant research earlier. One group that has been active lately includes Kim Kye-seong and Ramakrishna Suresh, who used CRISPR-Cas9 to conduct genome-scale knockout of the entire set of genes encoding ubiquitin-specific protease (USP) and screened USP3 as a deubiquitination enzyme (DUB) for the cell division cycle 25 oncoprotein (Das et al., 2020). This finding may help to screen target proteins of functional DUBs at the genomic scale. In addition to the above collaborator group, Hou Changjun has maintained a keen interest in CRISPR and cancer as an individual researcher in recent years.

### 3.3 Journals and institutions analysis

#### 3.3.1 Leading journals

**FIGURE 4:**
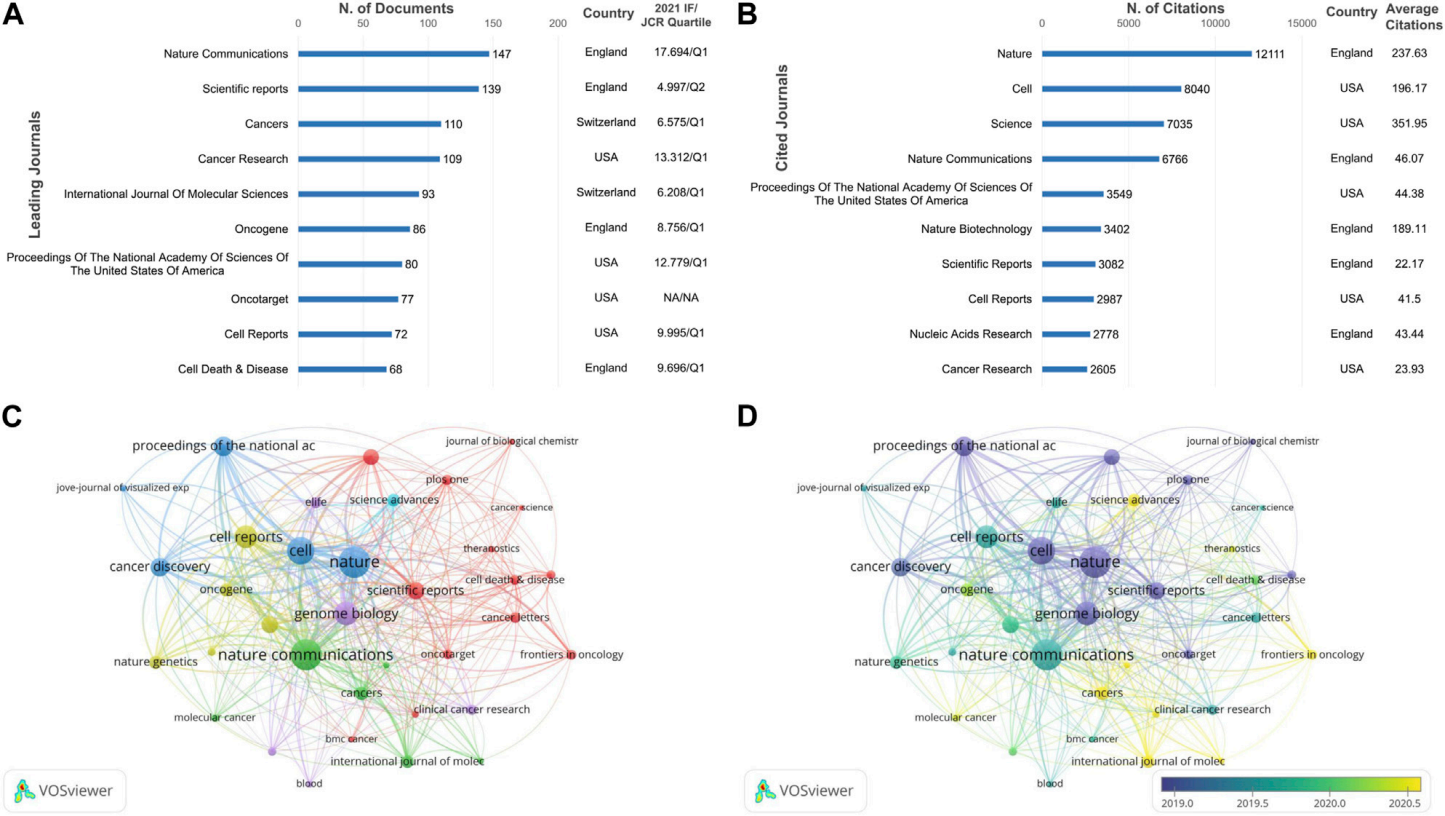
Bibliometric analysis of leading journals on CRISPR in cancer. **(A)** The top 10 published journals. **(B)** The top 10 cited journals. **(C)** Network visualization map of the leading journals. **(D)** Overlay visualization map of the leading journals.

In total, 645journals contributed 4,408 cancer-related articles involving CRISPR. The top 10 journals with the highest output and most citations are shown in Figures 4A, B, respectively.

As shown in Figure 4A, 2 journals published >130 papers, while the remaining journals had a smaller difference in the number of articles published. Among the journals, *Nature Communications* published the most articles (*n* = 147 [3.34%]) with the highest IF (17.694), and ranked fourth in citation counts. Among the top 10 journals, *Cancer Death & Disease* had the fewest number of articles (*n* = 68) with an IF/JCR quartile of 9.696/Q1 in 2021. As shown in Figure 4B, *Nature* had the most citations (*n* = 12,111), far surpassing the other top journals. *Science* had the greatest average citation number (351.95), followed by *Nature* (237.63). Among the 10 journals, one-half of the journals were published in the UK and the other half were published in the US.

As can be seen from the network visualization of the total link strength of 35 journals (Figure 4C), *Nature* and *Nature Communications* remained the leading journals. In addition, *Genome Biology*, *Cell Reports*, and *Cancer Discovery* also had high total link strengths, with IF/JCR quartiles of 18.010/Q1, 9.995/Q1 and 38.272/Q1, respectively. The overlay map (Figure 4D) demonstrated some emerging journals that were also of interest to researchers, such as *Cancers*, *Frontiers in Oncology*, and *International Journal of Molecular Sciences*. In conclusion, researchers can peruse different leading journals according to their needs.

#### 3.3.2 Collaboration of institutions

**FIGURE 5:**
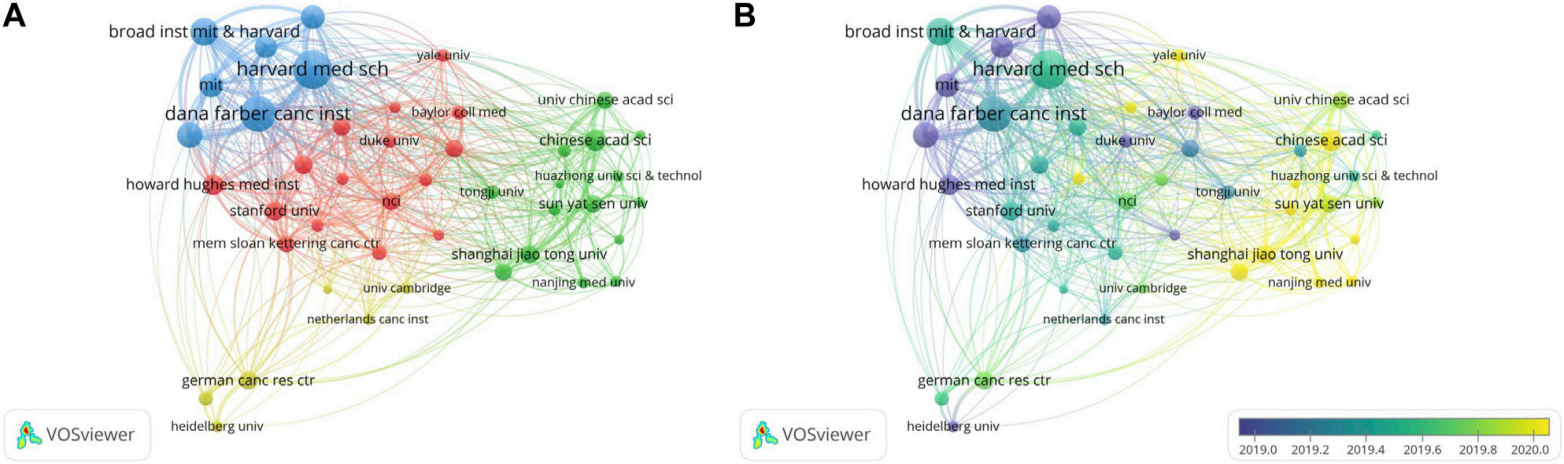
Co-authorship analysis of leading organizations on CRISPR in cancer. **(A)** Network visualization map of the leading organizations collaboration. **(B)** Overlay visualization map of the leading organizations collaboration.

We selected 44 affiliates with at least 42 articles for co-authorship analysis and produced network and overlay visualization maps of the collaboration (Figures 5A, B). As shown in Figure 5A the most collaborative research units were all in the blue cluster, including Harvard Medical School, Dana Farber Cancer Institute, and the Broad Institute of the Massachusetts Institute of Technology and Harvard. These three institutons were also among the top 5 most cited institutions, with >10,000 citations each. Harvard Medical School and Dana Farber Cancer Institute were also the top 2 institutions for publishing the most papers, with 237 and 149, respectively. One of these papers established Cre-dependent Rosa26 Cas9 knockin mice to overcome the Cas9 delivery difficulties, thereby enabling the application of Cas9-mediated genome editing *in vivo* (Platt et al., 2014). The study performed genome editing on these mice in lung tissues to study the dynamics of multiple mutations during tumorigenesis. The results demonstrated the potential of Cas9 mice to assist in the rapid screening of pathogenic gene mutations in a variety of biological and pathological processes (Platt et al., 2014). Another group of closely collaborating research institutions was in the green cluster, consisting of Chinese institutions and universities, such as the Chinese Academy of Sciences and Shanghai Jiao Tong University.

The overlay visualization (Figure 5B) shows that the most collaborative research teams started the study earlier than Chinese institutions. In the latest advance in the field, Chinese institutions reported an alternative strategy for enabling CRISPR-Cas9 delivery to cells or tissues (Wang et al., 2017). The strategy involves a non-viral delivery of Cas9 protein and single guide RNA (sgRNA) plasmid: a nanocarrier with gold nanoclusters (GNs) as the core and lipids as the shell. Cas9/sgRNA is delivered by modifying GNs with the HIV-1-transactivator of the transcription peptide. The study also demonstrated the ability of this strategy in delivering protein-nucleic acid hybrid agents for gene therapy by designing procedures to treat melanoma (Wang et al., 2017).

### 3.4 Countries analysis

#### 3.4.1 Leading countries

**FIGURE 6:**
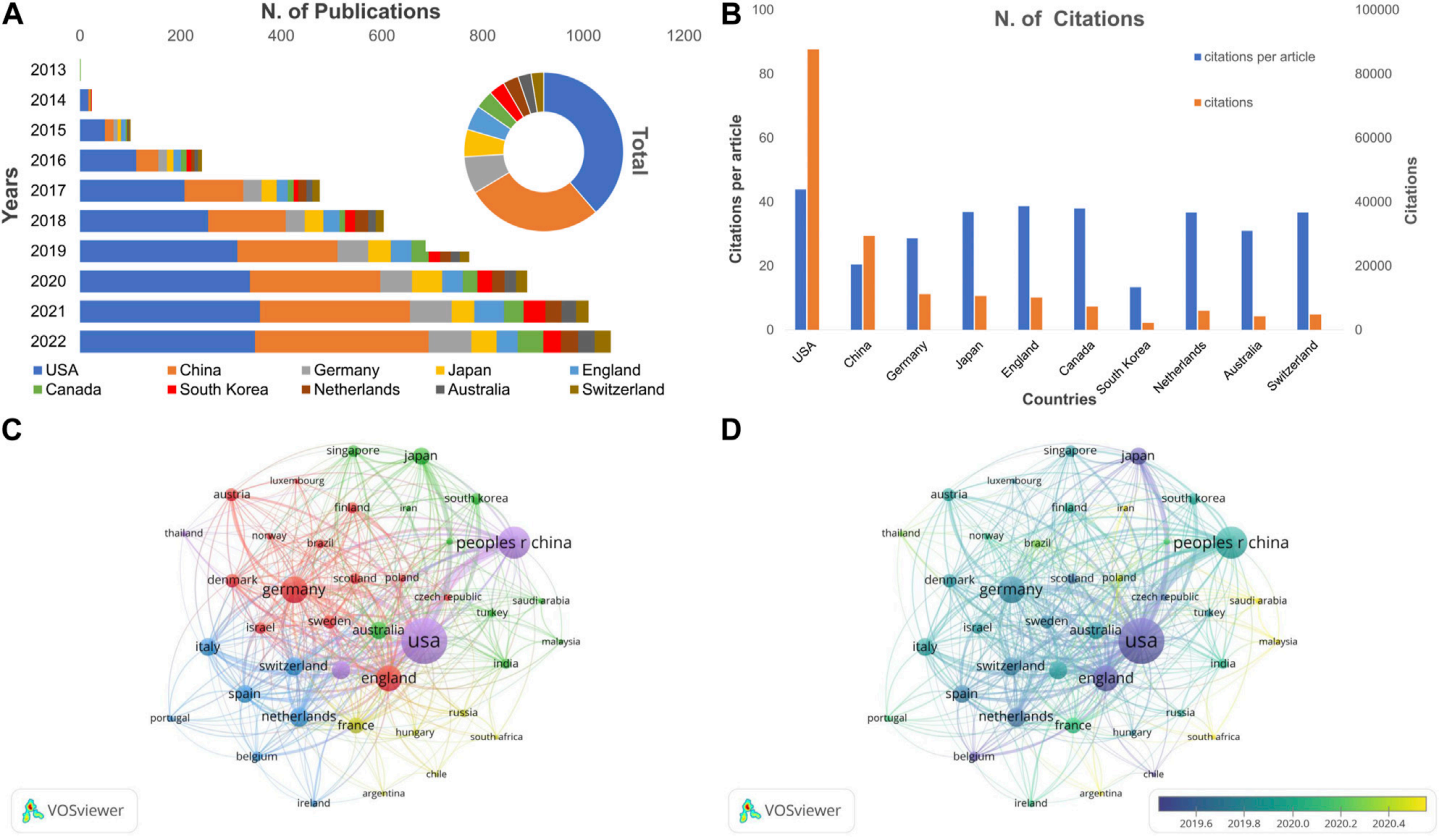
The analysis of leading countries/regions and co-authorships on CRISPR in cancer. **(A)** Annual and total publication volume for the top 10 countries. **(B)** Total citations, and average citations per article for the top 10 countries. **(C)** Network visualization map of the leading countries collaboration. **(D)** Overlay visualization map of the leading countries collaboration.

All the cancer publications involving CRISPR were distributed in 83 countries/regions. The United States ranked first with 1999 publications (45.34% of 4,408 papers), followed by People’s Republic of China (n = 1,436 [32.58%]), Germany (n = 391 [8.87%]), Japan (n = 288 [6.53%]) and England (n = 261 [5.92%]; Table 2). Among these countries, the United States and the People’s Republic of China published >1,000 articles, which are far more than any other country. The annual trends and total number of articles by assessing the 10 most productive countries are shown in Figure 6A. We noted that the United States had been the most active country in this area of research since 2013, followed by the People’s Republic of China, which surpassed the United States in 2021. Although other countries have been expanding annual research publications in recent years, the United States and the People’s Republic of China have long held the number 1 and 2 positions, respectively, with respect to total volume and number of annual papers.

**TABLE 2:** The top 10 most active countries/regions related to CRISPR in cancer.

Rank	Countries	Publications	Times cited	
			Total	Average per article
1	USA	1,999	87,757	43.90
2	China	1,436	29,436	20.50
3	Germany	391	11,218	28.69
4	Japan	288	10,630	36.91
5	England	261	10,098	38.69
6	Canada	192	7,285	37.94
7	South Korea	169	2,257	13.36
8	Netherland	163	5,984	36.71
9	Australia	139	4,308	30.99
10	Switzerland	132	4,848	36.73

As shown in Table 2, the six countries with the most published articles (United States, China, Germany, Japan, England, and Canada) were also the six countries with the greatest number of citations. The United States had greatest impact on area of research, with a total of 87,757 citations, which was greater than the other 9 countries combined. The finding changed with respect to the number of citations per article (Figure 6B; Table 2). The United States still ranked first among all countries on the list with 43.90 citations per article; however, the average per item (20.50 times) of China was lower than the other selected countries, except South Korea. South Korea had the lowest citation number (*n* = 2,257) and C:P ratio (13.36) among the most productive countries.

#### 3.4.2 Collaboration of countries

We selected 40 countries/regions with at least 9 publication frequencies for co-authorship analysis. As shown in Figure 6C, the United States ranked first with respect to the quantity of publications, citations, and total link strength, followed by the People’s Republic of China and Germany. Larger nodes and thicker linking lines implied that cooperation among leading countries had an important role in international exchanges. Countries in European cooperated more closely with each other than elsewhere. Netherlands only had 162 publications, but the Netherlands also cooperated extensively with other countries or regions, and the total link strength was higher than Canada, Japan, South Korea, and other countries.

Figure 6D shows an overlay visualization of co-authorship between countries/regions. The recently active countries/regions were distributed in the periphery, while the countries studied earlier were clustered in the center of the structure. Articles from Belgium, the United States, and Japan were published earlier than other countries. In contrast, cancer research involving CRISPR were conducted relatively late in South Africa, Saudi Arabia, and Malaysia. Moreover, the cooperative relationships between countries have declined in recent years and shifted to countries with weaker research efforts.

### 3.5 Co-citation and co-occurrence analysis

#### 3.5.1 Co-cited references and journals

**FIGURE 7:**
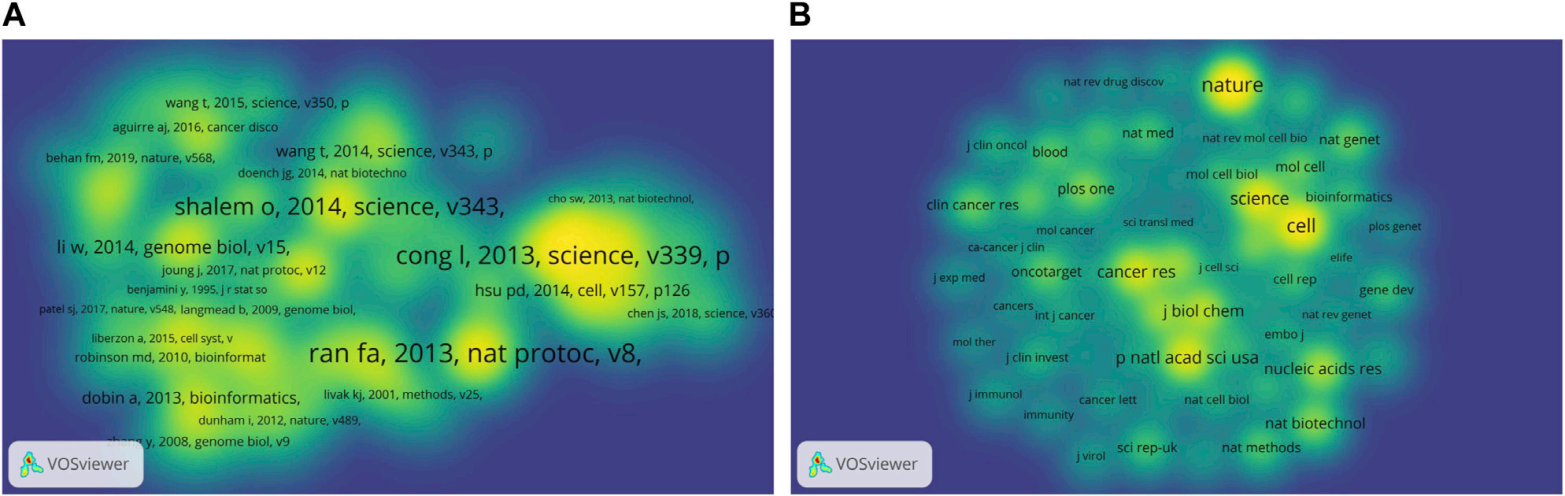
The bibliometric analysis of the co-citation on CRISPR in cancer. **(A)** Density visualization map of co-cited references. **(B)** Density visualization map of co-cited journals.

Co-cited references analysis could not only reveal revealed the change of in cancer research focus in the field, but also identified the core references of great significance for scientific decision-making in related fields (Chen et al., 2018; Tang et al., 2021). In this the current study, we used VOSviewer co-citation analysis to filter out the most co-cited references. In Figure 7A, 91,110 references were co-cited at least 50 times. We chose a density visualization map to make the their distribution clearer. Table 3 lists listed the top 10 co-cited references on involving CRISPR in or cancer. Half One-half of them the articles were published in Science, which demonstrated the journal emphasized both quantity and quality of publications. Moreover, and the corresponding author of the four articles was were Zhang Feng. , which reflected his outstanding academic and guidance ability.

**TABLE 3:** The top 10 most co-cited references related to CRISPR in cancer.

Rank	Article title	First author	Journal	Year
1	Genome engineering using the CRISPR-Cas9 system	Ran, F. Ann	Nat Protoc	2013
2	Multiplex genome engineering using CRISPR/Cas systems	Cong, Le	Science	2013
3	Genome-scale CRISPR-Cas9 knockout screening in human cells	Shalem, Ophir	Science	2014
4	Improved vectors and genome-wide libraries for CRISPR screening	Sanjana, Neville E	Nat Methods	2014
5	RNA-guided human genome engineering via Cas9	Mali, Prashant	Science	2013
6	A programmable dual-RNA-guided DNA endonuclease in adaptive bacterial immunity	Jinek, Martin	Science	2012
7	Gene set enrichment analysis: a knowledge-based approach for interpreting genome-wide expression profiles	Subramanian, Aravind	Proc Natl Acad Sci USA	2005
8	MAGeCK enables robust identification of essential genes from genome-scale CRISPR/Cas9 knockout screens	Li, Wei	Genome Biol	2014
9	Optimized sgRNA design to maximize activity and minimize off-target effects of CRISPR-Cas9	Doench, John G	Nat Biotechnol	2016
10	Genetic screens in human cells using the CRISPR-Cas9 system	Wang, Tim	Science	2014

The most co-cited reference, which is entitled “Genome engineering using the CRISPR-Cas9 system,”, was published by Ran F. Ann in Nature Protocols Nat Protoc in 2013 and had 553 co-citations. This study designed experiments and provided a set of tools to describe how to use Cas9 nuclease or nickase for genome editing in eukaryotic cells through homologous or non-homologous DNA repair pathways (Ran et al., 2013). The second most co-cited reference was is by Cong et al. (2013), which was published in Science in 2013, which and had 540 co-citations. Similar to the first one, this article had the same core value in this field. This paper revealed that short RNAs could guide guided Cas9 nucleases to precisely cleave endogenous genomic loci in human and mouse cells, and Cas9 could also promote promoted homology-directed repair with minimal mutagenic activity by converting conversion into a nicking enzyme (Cong et al., 2013). These studies demonstrated that the RNA-guided nuclease technology was programmable, easy to program and laying the foundation for the application of CRISPR-Cas in cancer research. had wide application prospects. Another reference with more than >400 co-citations was published by Shalem Ophir, O in 2014, which was also the most cited article, indicating the great influence and authority of the research.

It was worth noting that the majority most of the top co-cited studies literatures were published after 2012, except the Subramanian’s Aravind article that was published in 2005. This article was not related to the field of CRISPR and caner, but it developed a Gene Set Enrichment Analysis tool. It was The tool is an analytical method for analyzing molecular profiling data, thus allowing researchers to focus on gene expression data at the level of gene sets (Subramanian et al., 2005). Articles with high co-citations reveal the significance of the work revealed the hotspots and directions in the field, and the number of citations disclosed their significance, so the relevant references or journals are were worth reading and studying carefully.

As can be seen from the density visualization map of journals (Figure 7B), Nature, Cell, and Science are were the journals with the highest number of co-citations, as well as the journals with the highest number of citations. The journal Proceedings of the National Academy of Sciences of the United States, which had an IF of 12.779 in 2021, ranking fourth fourth in number terms of co-citations with a relatively low number of publications (n = 80 [1.82%] 84 articles, 1.75%) and citations (n = 3,549 3,860 times) compared to the top 3 journals three. Combined with the previous analysis of leading journals, Nature and it is the Nature sub-journals had have made significant contributions to CRISPR in cancer research, while being authoritative.

#### 3.5.2 Keywords analysis

**FIGURE 8:**
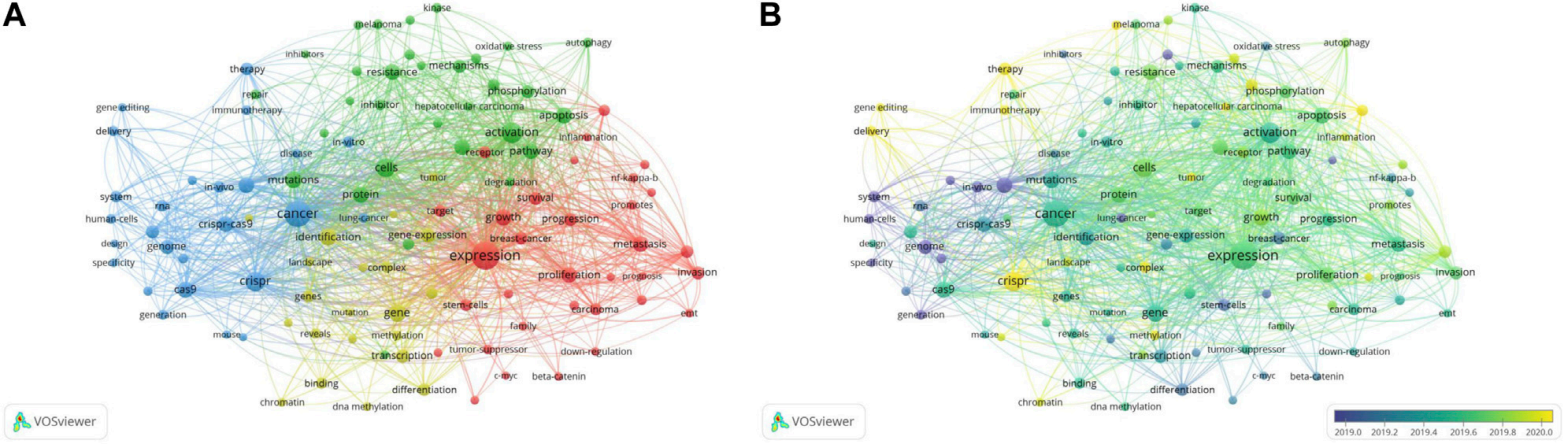
Co-occurrence analysis of all keywords on CRISPR in cancer. **(A)** Network visualization map of co-occurrence of the high frequency keywords. **(B)** Overlay visualization map of co-occurrence of the high frequency keywords.

Keyword co-occurrence analysis was a complementary tool that enriches understanding of relevant research hotpots and trends, and predicts future research in the field (Donthu et al., 2021). We selected the “all keywords” unit of the occurrence analysis to display the network and overlay visualization map. The threshold was set as > 50 occurrences and 107 high-frequency keywords were selected from 15,032 keywords (Figures 8A, B).

Figure 8A showed 4 clusters of the keyword network, as shown below: Cluster 1, “expression of inflammatory molecules in the development and progression of cancer” (red), included expression, beta-catenin, breast cancer, carcinoma, proliferation, progression, and inflammation; Cluster 2, “mechanisms of oxidative stress on cancer development” (green), including oxidative stress, pathways, apoptosis, mutations, activation, and inhibitor; Cluster 3, “genome editing with CRISPR in cancer” (blue), consisting of cancer, CRISPR-Cas9, gene editing, human cells, mouse, and immunotherapy; and Cluster 4, “role of DNA methylation of genes in cancer” (yellow), with DNA methylation, gene-expression, transcription, identification, and differentiation. Cluster 1 and 2 were shown to be closely related to the study of cancer occurrence, promotion, and progression. Cluster 3 was relevant to the CRISPR-Cas system with respect to basic research and therapeutic applications. Cluster 4 was associated with cancer gene modification. Although the general direction of these clusters was summarized, some key words may require review of the article to understand the meaning. Among the high frequency keywords, “expression,” “cancer,” and “activation” ranked as the top three for number of occurrences times and total link strength. Therefore, use of CRISPR in cancer research focused onthese three directions.

The overlay visualization map (Figure 8B) showed that early research was limited to cancer stem cells and several types of cancer, then expanded to advances in basic cancer research and clinical applications of CRISPR-Cas-mediated gene editing technology. Over time, research hotspots had been developed and included related fields; however, new research priorities in this field have not clearly emerged. The light-yellow color of the keyword ‘crispr’ indicated its later average age, which may be because CRISPR-related research had been ongoing compared to other directions. Based on this map, we inferred that related therapeutic research and optimization protocols, such as immunotherapy and delivery methods, may be the next research trend.

## 4 Research hotspots and frontiers

Based on the co-citation references and co-occurrence keywords analysis, we identified disease modeling, novel target discovery, and cancer immunotherapy innovations as research priorities and frontiers for CRISPR.

### 4.1 CRISPR-cas system and genetic scissors

CRISPR-Cas systems exists widely in a broad range of bacterial species and provides rich functional versatility and efficiency for genome editing in eukaryotic cells (Adli, 2018; Hendriks et al., 2020). This gene editing technique, derived from the bacterial immune system, is widely exploited in the type-II CRISPR-Cas9 system of *Streptococcus pyogenes*. The type-II CRISPR system generally consists of CRISPR RNA (crRNA), trans-activating crRNA (tracrRNA), and Cas9 protein. The overall immune response relies on the specific recognition of the protospacer adjacent motif by crRNA (the tracrRNA duplex and the cleavage of targeted DNA sequences by Cas9 protein) (Garneau et al., 2010; Sternberg et al., 2014). To be used in manipulating genomes, the CRISPR system is reprogrammed by combining crRNA with tracrRNA to form sgRNA, which directs Cas9 endonuclease to perform sequence-specific DNA double-strand breaks (DSBs) in target DNA (Gasiunas et al., 2012; Jinek et al., 2012). Subsequently, DSBs can be exploited for genetic engineering purposes through two different repaired pathways (Cho et al., 2013): homology-directed repair or more frequently non-homologues end joining; followed by the introduction of precise modifications or small indels into the target sequence (Rouet et al., 1994; Doudna and Charpentier, 2014; Yeh et al., 2019).

Due to the simplicity and efficiency of gene manipulation and the programmability of sgRNA, the CRISPR-Cas system has become a widely used method for mammalian genome editing (Mali et al., 2013). Because cancer is a genetic disease caused by cumulative genetic/epigenetic aberrations, the potential of this genome editing tool for basic research and clinical applications is particularly evident in cancer research and therapeutics (Chen et al., 2019).

### 4.2 CRISPR-cas for cancer modeling

To identify driver genes and interrogate gene functions in tumorigenesis, progression, and maintenance, the generation of genetically-defined models is a core approach. The CRISPR-Cas genetic engineering systems provides rapid, simple, and accurate disease models for studying the genetic determinants of cancer and validating drug targets in immuno-oncology.

#### 4.2.1 *In vitro* models

With the efficiency and capability of CRISPR tools, it is feasible to generate *in vitro* or *in vivo* models of cancer with the characteristics of human disease. In addition to performing pharmacologic studies and verifying the role of identified genes in cancer cell lines, another common *in vitro* model, the three-dimensional organoid, has been genome-edited to study tumor biology (Wanzel et al., 2016; Lin et al., 2017). A recent studyused CRISPR-based genome engineering to establish primary human gastric cancer organoid models a TP53 mutation and AT-rich interactive domain 1A (ARID1A) knockout (Lo et al., 2021). ARID1A knockout organoids clearly elucidated the mechanism and role of ARID1A deletion in oncogenic transformation of gastric epithelium in the absence of TP53 (Lo et al., 2020). Primary human organoids accurately mimic the *in vivo* biology of native cancer. This finding has important implications for personalized anti-cancer medicine and precision-targeted drug screening (Lo et al., 2020), as well as for the discovery of genetic and epigenetic markers and prognosis based on relevant hallmarks (Hay et al., 2014).

#### 4.2.2 *In vivo* models

In addition to studying cancer-related events in *vitro* models, CRISPR enables the rapid creation of complex and precise animal disease models. Among these models, the most commonly used is the *in vivo* KO mouse model. CRISPR technology facilitates the development of transgenic models using engineered mouse embryonic stem cells (Wang et al., 2013), but also the introduction of all CRISPR components into tissues to induce and recapitulate carcinogenesis caused by mutations in somatic cells (Winters et al., 2018). For the latter model construction approach, targeting and delivering CRISPR components directly *in vivo* leads to a more rapid generation of diverse cancer models with complicated cancer genotypes than *ex vivo* manipulation and transplantation of cultured cells (Katti et al., 2022).

Another animal model was created by surgically-transplanting xenograft from a patient into immunodeficient mice. This patient-derived xenograft (PDX) cancer model may maintain the histologic heterogeneity of the patient’s tumor (Chandrasekaran et al., 2022). Researchers induced immune deficiency in Sprague-Dawley rats by knockout of Rag1, Rag2, and Il2rg, and established a PDX model of squamous lung cancer using this novel rat model. The grafts recapitulated the histopathological characteristics of the primary tumor in several passes (He et al., 2019). Overall, CRISPR animal cancer models have played a key role in revealing the basics of tumor initiation, maintenance, and progression. In addition, CRISPR animal cancer models have become faithful models for testing various anti-cancer agents, as well as mechanisms of detecting drug resistance using CRISPR screening (Chen et al., 2012; Sánchez-Rivera and Jacks, 2015).

### 4.3 CRISPR-cas for target screening

With the help of improved sgRNA libraries, CRISPR-Cas knockout screening technology facilitates the interrogation of cancer-related gene function in various cancer models to discover new therapeutic targets (Sanjana et al., 2014; Sanson et al., 2018).

#### 4.3.1 *In vitro* screening

Both genome-wide and focused loss-of-function CRISPR screening have been successfully adapted to facilitate the identification of genotype-specific vulnerabilities in cancer cell lines. Several high-throughput CRISPR genetic screening studies of genome-scale lentiviral sgRNA libraries have been established in a variety of cell types (Wang et al., 2014; Wang et al., 2015). In these screening studies, cultured cells can be transfected and knocked out with various types of CRISPR libraries (Cas9+sgRNA) and incubated *in vitro* under the desired experimental conditions (Wang et al., 2014; Sanson et al., 2018). Subsequent selection assays resulted in the enrichment or depletion of sgRNAs in the library depending on the targeting genes of candidate tumor suppressor genes (Chow et al., 2017) or drug sensitivity genes (Shi et al., 2015), respectively. Next-generation sgRNA sequencing analysis will identify and evaluate “hit” events to recover known targets or discover unknown targets for validation (Yin et al., 2019). In addition to genome-wide libraries, the researchers have also developed specific sgRNA libraries. These libraries are able to target kinases/proteins involved in genetic regulation and mediate CRISPR–Cas9–based epigenomic regulatory element screening to improve high-throughput screening of regulatory element activity at the native genomic scale (Klann et al., 2017).

Advanced screening studies have been conducted with both types of libraries to identify genes implicated in sensitivity to therapeutic agents, such as a BRAF inhibitor (vemurafenib) (Shalem et al., 2014) and a nucleotide analogue (6-thioguanin) (Sanson et al., 2018), as well as to reveal novel candidate genes involved in drug resistance using the drug perturbation method (Krall et al., 2017). The knock-in screening has also been applied to identify cancer predisposition mechanisms and potential therapeutic targets. Specifically, the gain of WW domain function containing protein 1 as a cancer susceptibility gene triggers phosphate and tension homology gene deletion on chromosome 10 ubiquitination and inactivation, as well as phosphoinositide 3-kinase signaling hyperactivation (Lee et al., 2020).

#### 4.3.2 *In vivo* screening

In addition to screening in cultured cell lines, CRISPR-mediated screening studies have been conducted *ex vivo* and *in vivo*, as in the animal model construction described above. *ex vivo*, The genome-wide libraries were modified in a cell pool, then transplanted into recipient mice for *ex vivo* screening. In this way, several studies have isolated tumors formed from modified and transplanted cells. Genetic characteristics were screened to identify the effect of different genetic aberrations on tumor development or treatment response (Braun et al., 2016; Katigbak et al., 2016; Kodama et al., 2017). In addition, *ex vivo* screening was used to identify metastasis regulators in non-small cell lung cancer (Chen et al., 2015). Using a similar approach, modified cancer cells were used for xenografts to identify genes that mediate the response to anti-cancer immunotherapy (Manguso et al., 2017).

Unlike *ex vivo* modification, *in vivo* CRISPR screening has been performed by direct introduction of sgRNA mutant libraries into non-transformed tissues through adeno-associated virus or hydrodynamic injection (Weber et al., 2015). A representative study delivered a genome-wide AAV sgRNA library to the mouse brain model that could induce Cas9-expression to identify tumor suppressor genes and reveal a subset of cancer drivers in resultant glioblastomas (Wang et al., 2015). Genome-scale CRISPR screening has accelerated the discovery of novel drug targets in cancer through a range of creative approaches (Tzelepis et al., 2016).

### 4.4 CRISPR for cancer immunotherapy

Cancer immunotherapy is an emerging method of cancer therapy that has achieved clinical benefits in a variety of cancers by generating a highly specific and powerful immune responses to attack tumors (Khalil et al., 2016; Alok et al., 2022); however, due to the variable therapeutic efficiency of this new therapy, CRISPR technology has been applied to improve the efficacy and safety for cancer immunotherapy.

CRISPR can be used to inactivate immune checkpoint genes in primary T cells, such as genes encoding programmed cell death 1 (PD-1) and cytotoxic T lymphocyte antigen 4. Moreover, the discovery of immune checkpoints can be performed through CRISPR-based screening, such as the discovery that deletion of the tyrosine protein phosphatase, PTPN2, in melanoma cells sensitizes mice to PD-1 inhibition (Weber et al., 2015).

In addition to immune checkpoints, CRISPR technology has potentially revolutionized adoptive T cell therapy (ACT). ACT is an immunotherapy with a robust anti-tumor response that manipulates T cells *ex vivo* to increase anti-cancer potency. This manipulation includes the purification and expansion of tumor-infiltrating lymphocytes, as well as the two main therapies currently under investigation (targeted insertion of chimeric antigen receptors [CARs] and engineered T cell receptor [TCRs]) (Maus et al., 2014). The great potential of CAR-T cells in immunotherapy has been confirmed in a variety of clinical trials (Brudno and Kochenderfer, 2018; D’Aloia et al., 2018), such as the complete response induced by CD-19 targeted CAR-T cells in patients with refractory B cell acute lymphoblastic leukemia (D’Aloia et al., 2018).

**FIGURE 9:**
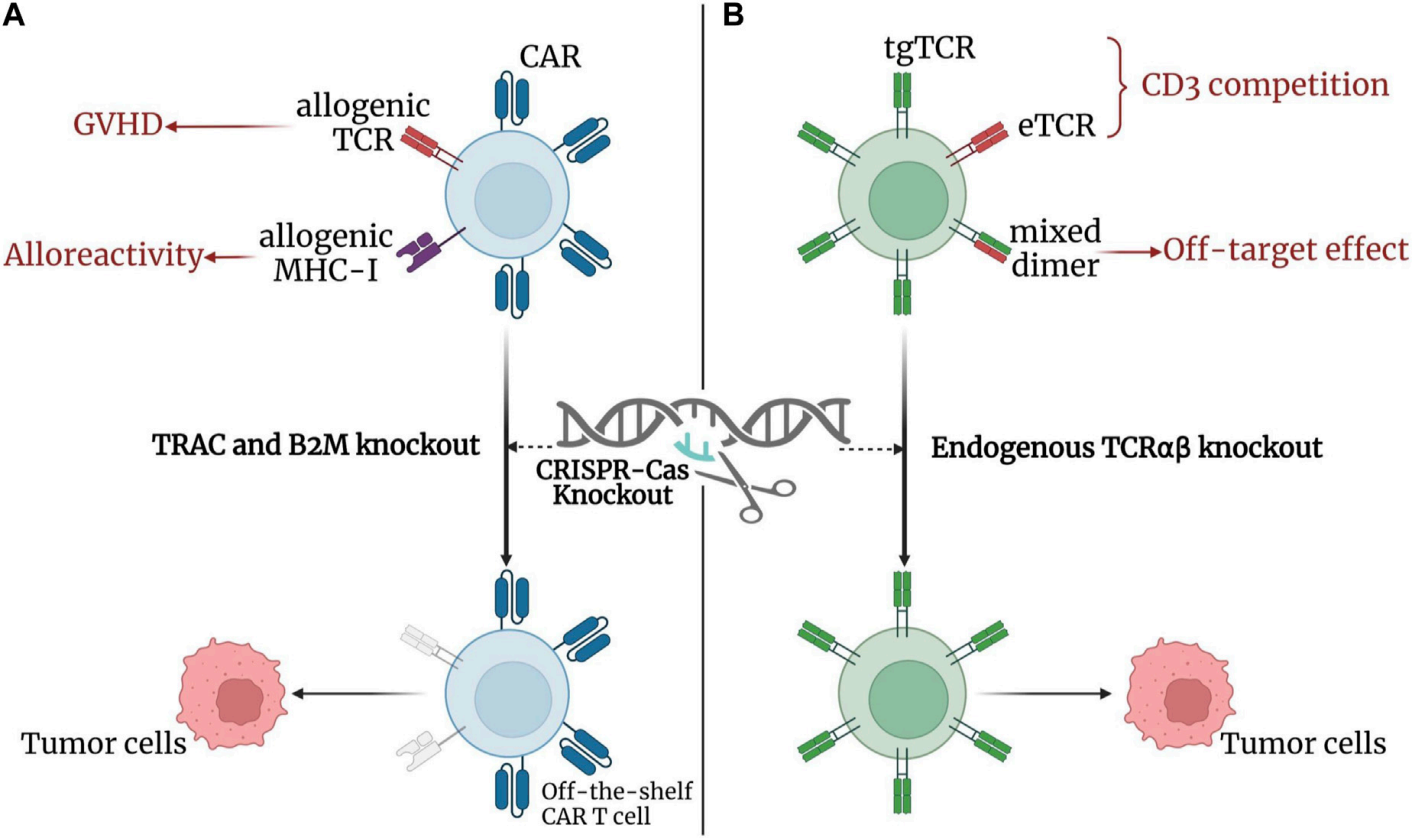
CRISPR-Cas in adoptive T cell therapy. **(A)** CRISPR-Cas in CAR T-cell therapy: knockdown of TRAC and B2M using CRISPR reduces the risk of GVHD and limits the alloreactivity of allogenic T cells, respectively, thus improving the anti-tumor efficacy and safety of CAR-T cell therapy. The findings contributes to the establishment of off-the-shelf CAR-T cells. **(B)** CRISPR-Cas in TCR T-cell therapy: knockdown of endogenous TCRαβ with CRISPR reduces the binding competition and mismatch between tgTCRs and eTCRs, thus reducing off-target effects and increasing the surface expression of tgTCRs. Created with BioRender.com.

Despite the success of CAR-T cell therapy, some limitations remain, such as the cost and complexity of autologous T-cell manipulation (Fix et al., 2021), the graft-versus-host disease (GVHD) caused by allogenic T cells (Ghosh et al., 2017), and the low efficacy of CAR-T cell therapy in solid tumor treatment (Lamers et al., 2006). Genome-editing platforms, especially CRISPR, have emerged as powerful tools to overcome these limitations and improve the anti-tumor efficacy and safety of CAR-T cell therapy (Figure 9A). The CRISPR system can target CAR to the TCRα constant (TRAC) (Eyquem et al., 2017) or delete β_2_-microglobulin (β_2_M) (Ren et al., 2017) to silence the TCR or HLA-I of allogenic T cells. This effect helped reduce the risk of graft reactivity and limit rejection of allogenic T cells, thus paving the way for the use of off-the-shelf CAR-T cells (Liu et al., 2017). Additionally, CRISPR-Cas9-mediated PD-1, TRAC, and β_2_M polygenic disruption of CAR-T cells resulted in enhanced anti-tumor activity in preclinical models of human glioblastoma (Choi et al., 2019). This finding suggests a role for CRISPR-mediated PD-1 disruption in addressing CAR-T cell therapy failure in solid tumors due to immunosuppressive tumor microenvironment and CAR-T cell exhaustion.

TCR-T cell therapy, like CARs, T cells can be modified with defined TCRs in response to specific tumor antigens. Moreover, TCRs can recognize intracellular proteins (Morris and Stauss, 2016), which expands the range of tumor antigen recognition for TCR-T cells therapy and allows TCRs to target cancer-mutated genes (Morris and Stauss, 2016). This TCR-T cell therapy property shows potential against solid tumors; however, the mismatch and competition between endogenous TCRs (eTCRs) and transgenic TCRs (tgTCRs) limit the frequency of tgTCR expression in edited T cells (Bendle et al., 2010; van Loenen et al., 2010). To address the resulting off-target effects, CRISPR technology (Figure 9B) has been used to improve expression and enhance recognition by knocking out endogenous TCRαβ (Morton et al., 2020). The combination of multiple CRISPR-Cas9 editing of TRAC, TCRβ constant, and PDCD1 with introduction of a cancer-specific TCR transgene (NY-ESO-1) improved anti-tumor immune responses and reduced TCR mismatches in a phase I human clinical trial (Stadtmauer et al., 2020). In addition to developing more effective and safer cancer immunotherapy with TCR-T cell therapy, CRISPR can also interferes with immune checkpoint genes. TCR-T cell therapy can overcome gene suppression and enhance anti-tumor immune responses, and its application in human antigen-specific cytotoxic T lymphocytes can improve the anti-tumor function of PD-1 deleted cells (Zhang et al., 2018).

Other than investigating the above two prevailing therapies, the potential and applications of other immune cellular therapies are beginning to emerge. Specifically, natural killer (NK) cells are ideal candidates due to their direct and non-antigen-specific killing effect on cancer cells. Pomeroy et al. (2020) performed high-efficiency gene editing of primary NK cells using CRISPR-Cas9, and reported the improved impact of NK inhibitory signaling molecules (ADAM17) and PD-1 gene knockout on NK cell-based cancer immunotherapy. The study demonstrated the enhanced antibody-dependent cytotoxicity of CRISPR-edited NK cells and provided a universal approach for generating engineered primary NK cells for cancer immunotherapy (Pomeroy et al., 2020). In addition to NK cells, the researchers extended CRISPR to macrophage-based immunotherapy. Wang et al. (2021) utilized CRISPR knockout screens and several analyses to identify the E3 ubiquitin ligase, Cop1, as a regulator of macrophage infiltration and a target for improving the efficacy of cancer immunotherapy (Wang et al., 2021). Another study demonstrated that knockout of signal regulatory protein-α in macrophages by CRISPR prevents immune escape and enhances phagocytosis of tumor cells (Ray et al., 2018). Based on such progressions, we anticipate that CRISPR-Cas technology will have more applications in cancer immunotherapeutics and continue to mature in the coming years.

## 5 Strengths and limitations

In this study we performed a systematic and comprehensive analysis of the global scientific literature on CRISPR as related to cancer. Compared with traditional literature reviews, the bibliometric analysis and application of VOSviewer software improved objectivity and comprehensiveness; however, there were some notable limitations in this study. First, our analysis only collected documents from the WoS database, resulting in bibliography omissions. Second, the selected literature only included articles published in English from 2013 to 2022, which may cause selection and time bias. Third, the search strategies omitted searches for main text and some articles that contained only one aspect of keywords or no keywords. Finally, some recently published high-quality studies may not receive enough attention because they were cited less frequently than classical papers, which inevitably leads to literature omission.

## 6 Conclusion

This study systematically summarized the global cancer publications involving CRISPR and investigated the distribution and collaboration of scientific outputs through bibliometric and visual analysis. The analysis revealed a steady upward trend in the number of publications, with the United States and the People’s Republic of China making substantial contributions to the field. The journal with the most publications was *Nature Communications*, while the journal with the most citations was *Nature*. Li Wei and Zhang Feng were the authors with the most publications and citations, respectively. Collaboration among research constituents should be expanded and strengthened to promote academic progress and fill research gaps in this field. We hoped that countries could provide platforms for exchanges and cooperation between researchers and institutions. CRISPR had been at the center of interest in cancer modeling and target discovery. Immunotherapy using CRISPR-Cas system had provided future research directions and may facilitate precise medicine for cancer patients through genetically-defined models. Collectively, our study combined an analysis of overall CRISPR research and a review of specific CRISPR cancer applications, summarizing and predicting research directions that further accurately guide cancer researchers.

## Data Availability

The original contributions presented in the study are included in the article/Supplementary Material further inquiries can be directed to the corresponding authors.

## Abbreviations

CRISPR, Clustered regularly interspaced short palindromic repeats; Cas, CRISPR associated system; WoS, Web of science; IF, Impact factor; JCR, Journal citation reports; USP, Ubiquitin-specific protease; DUB, Deubiquitinating enzyme; AAV, adeno-associated virus; sgRNA, single guide RNA; GNs, gold nanoclusters; crRNA, CRISPR RNA; tracrRNA, Trans-activating crRNA; DSBs, Double strand breaks; ARID1A, AT-rich interactive domain 1A; PDX, Patient-derived xenograft; PD-1, Programmed cell death 1; ACT, Adoptive T cell therapy; CAR, Chimeric antigen receptors; TCR, T cell receptor; GVHD, Graft-versus-host disease; TRAC, TCRα constant; β2M, β2-microglobulin; eTCRs, Endogenous TCRs; tgTCRs, Transgenic TCRs; NK, natural killer.
