# Supplementary material for: Knowledge mapping and current trends of global research on CRISPR in the field of cancer
Source: Front Cell Dev Biol. 2023 May 2;11:1178221. doi: 10.3389/fcell.2023.1178221 (PMC10185797; doi:10.3389/fcell.2023.1178221)
Supplement: Supplementary file 4 [file DataSheet1.DOCX]

Knowledge mapping and current trends of global research on CRISPR in the field of cancer

Han Liu^1,#^, Zongwei Lv^1,#^, Gong Zhang^1,#^, Xia Wang^1^, Yuan Wang^2,*^, Kefeng Wang^1,*^

^1^Department of Urology, Shengjing Hospital of China Medical University, Shenyang 110004, China

^2^Department of General Surgery, Shengjing Hospital of China Medical University, Shenyang 110004, China

**# These authors contributed equally to this work.**

***Correspondence to：**Kefeng Wang, address: #36 Sanhao Street, Heping District, Shenyang, Liaoning, China. Email: [wang.kefeng@hotmail.com](mailto:wang.kefeng@hotmail.com). Tel: +86 18940254849, Fax: +86 24 31939077. Yuan Wang, address: #36 Sanhao Street, Heping District, Shenyang, Liaoning, China. Email: wangyuan19830711@126.com. Tel: +86 18940256118, Fax: +86 24 31939077.

**Abstract**

**Background**: Gene editing tools using clustered regularly interspaced short palindromic repeats (CRISPR) and CRISPR-related systems have revolutionized our understanding of cancer. The purpose of this study was to determine ~~investigate~~ the distribution, collaboration, and direction of cancer research using CRISPR. ~~research outputs on CRISPR in the field of cancer.~~

**Methods**: Data from the Web of Science (WoS) Core Collection database were collected from ~~4,814 papers~~ 4408 cancer publications related to CRISPR ~~in cancer~~ from 1 January ~~1,~~ 2013 ~~2012~~ to 31 December ~~31,~~ 2022. The obtained data were analyzed using ~~through~~ VOSviewer software for citation, co-citation, co-authorship, and co-occurrence analysis.

**Results**: ~~Over the past decade,~~ The number of annual publications has ~~have~~ grown steadily over the past decade worldwide. The United States~~, followed by China,~~ was shown, by far, to be the leading source of ~~country on CRISPR in~~ cancer ~~, with far more~~ publications, citations, and collaborations involving CRISPR than any other country, followed by China. Li Wei (Jilin University, China), and Harvard Medical School (Boston, MA, USA) were the author and institution ~~organization~~ with the most publications and ~~the most~~ active collaborations, respectively. The journal with the most contributions was *Nature Communications* (n=147) ~~Scientific Reports (156 publications),~~ and the journal with the most citations was *Nature* (n=12,111 ~~11,723 citations~~). The research direction of oncogenic molecules, mechanisms, and cancer-related gene editing was indicated based on ~~by~~ keyword analysis.

**Conclusions**: ~~Our~~ The current study has provided ~~provides~~ a comprehensive overview of ~~the~~ cancer research highlights and future trends of CRISPR, combined with a review of CRISPR applications in cancer to summarize and predict research directions and provide guidance to researchers.  ~~in cancer, which may further facilitate the basic research and clinical applications in this field.~~

**Keywords:** Bibliometrics; CRISPR; Cancer; Cas; Immunotherapy

**1. Introduction**

Cancer is a complicated and ~~refractory~~ hereditary disease~~, which~~ that has attracted worldwide attention. Studies have shown that tumorigenesis is comprised of ~~comprises~~ cumulative somatic mutations and epigenetic aberrations of oncogenes and tumor suppressor genes [1,2]. Cancer kills one in six people worldwide~~,~~ and threatens ~~threatening~~ thousands of lives [3–5]. ~~Over the past 20 years,~~ With the discovery of high-throughput sequencing technology, a large number of genes related to the occurrence and development of cancer have been discovered over the past 20 years [6]. ~~Gene editing technology has great potential for cancer treatment by modifying gene expression and correcting mutations, and has become a research hotspot in recent years.~~ Gene editing technology has facilitated these advances in cancer research [7] and further helped to identify therapeutic targets [8].

~~So far,~~ Several techniques have been applied to achieve gene editing. Since the discovery of RNA programmability and mammalian cell adaptation [9,10], ~~the~~ clustered regularly interspaced short palindromic repeats (CRISPR) technology and CRISPR-associated ~~(Cas)~~ system (Cas) ~~systems~~ ~~technology has~~ have come to be recognized as a revolutionary gene editing toolkit [11]. The CRISPR-Cas ~~system~~ is an RNA-mediated adaptive immune system that provides acquired immunity against invading viruses and phages in bacteria and archaea [12,13]. Because of the ~~its~~ advantages of a simple and rapid design, the CRISPR-Cas ~~this system~~ has been widely used, especially in cancer biology research [14]. Gene editing tools have had a major impact on ~~the field of~~ cancer biology and are emerging as a promising approach to cancer diagnosis and treatment.

Given the rapid development of CRISPR in cancer research, various detailed reviews are emerging ~~springing up~~. Although some conclusions are clear and insightful, there is a lack of complete and macro-quantitative research. In contrast, bibliometrics is a new statistical and mathematical approach used to analyze scientific outputs, thus providing investigators with both qualitative and quantitative characteristics [15,16]. ~~It~~ Bibliometrics allows large-scale, objective summaries of existing literature in an area of research ~~a field~~, thus enabling researchers to clarify current research trends in ~~and~~ core areas [15,17].

~~Based on this~~ Therefore, we conducted a systematic investigation of CRISPR-related scientific achievements in cancer from 2013-2022 ~~2012 to 2022~~ to identify ~~the~~ current research trends in cancer biology ~~this field~~. Then, various bibliometrics and visual indicators were used to reveal ~~their~~ the relevant contributions, influence, and co-authorship structure. ~~Our~~ The current study ~~summarized~~ summarizes the development of CRISPR in basic cancer research and the clinical application~~s in cancer~~.

**2. Materials and methods**

**2.1 Data source and collection**

We retrieved the world’s ~~global~~ publications that utilized ~~on~~ CRISPR in ~~the field of~~ cancer research from the Web of Science (WoS) Core Collection database (Clarivate). The detailed data retrieval and exclusion process of this study are provided in **Figure 1**. The search strategies in the advanced section were as follows: TS = (CRISPR OR "clustered regularly interspaced short palindromic repeats") AND TS = (cancer OR carcinoma OR malignant*). The data spanned from 1 January 2013 ~~2012~~ to 31 December 2022. The type of publication was limited to “article” ~~and~~ only published in English. ~~Finally, we~~ Then, we excluded irrelevant papers and finally obtained a sample of 4408 ~~4,814~~ articles for analysis. The relevant records were exported to VOSviewer (version 1.6.18; Leiden University) as a plain text file in “full record and cited references” format.

**2.2 Data analysis and visualization**

The VOSviewer software ~~(version 1.6.18)~~ was selected as the ~~a~~ primary tool for comprehensive analysis and network construction ~~networks constructing~~ to visualize and inspect large bibliometric networks using ~~through~~ graphical presentations [18]. We created visualization maps to analyze the intellectual interactions and structural connections among research constituents through different bibliometric functions. The analysis results are ~~were~~ presented in network, overlay, and density visualization~~,~~ consisting of items/clusters in different colors and lines between them. The size of circles was positively correlated with the contributions of research constituents. The ~~particular~~ cluster color ~~of clusters~~ indicated the order of the cluster number ~~of different clusters~~. In addition, the ~~shades of~~ circle color shade in the overlay map reflected the proximity of the year to the article. The lines between the items demonstrated the relationship and strength of the ~~their~~ cooperation.

The WoS “analyze results” and “citation report” functions ~~of WoS~~ were used to perform basic publication and citation statistics, including publications over time, the times cited, and the average citations per article ~~H-index~~. The impact factor (IF) and quartile ranking were collected from the 2021 edition of *Journal Citation Reports (JCR)* ~~2021 edition~~. Microsoft Excel 2019 was ~~were~~ used to draw and analyze the publications, ~~and~~ citations trends, and major constituent ~~constituents~~ (authors, journals, and countries/regions) distribution. Microsoft PowerPoint 2019 was used ~~applied~~ to draw the flowchart depicting ~~of~~ literature screening.

**3. Results**

**3.1 Overview of annual publications and citations**

The number of publications and citations per year or per research constituent were the most prominent measures of performance analysis, representing productivity and impact, respectively [16]. **Figure 2** depicts ~~depicted~~ the annual trend of publications, ~~;~~ the distribution of citations, and the ~~their~~ function-fitting curves. From 2013-2022 ~~2012 to 2022~~, the number of cancer-related publications involving ~~on~~ CRISPR ~~in the field of cancer~~ increased from 1 to 4408 ~~4,814~~. The increase in the number of articles was more pronounced in 2016-2017 than ~~in~~ other years, while a slight downward trend occurred ~~was seen~~ in 2021-2022. The growing trend indicated that CRISPR use in cancer research has attracted significant attention over the past 11 ~~eleven~~ years.

In addition, a total of 4408 ~~4,814~~ articles were cited 128,834 ~~132,642~~ times, with an average of 29.23 ~~27.55~~ citations per article. Both the exponential growth in citation number ~~numbers~~ and the curve-fitting upward trend demonstrated the global interest and cancer research potential in using CRISPR ~~this field~~. In summary, we deduced that CRISPR would receive more attention and have greater cancer research ~~future~~ prospects in the future ~~field of cancer~~.

**3.2 Authors analysis**

**3.2.1 Leading authors**

Greater ~~More~~ than 30,000 researchers have ~~had~~ contributed to CRISPR-related cancer research ~~in cancer~~. **Table 1** lists ~~listed~~ the top 10 most productive authors, along with the ~~their~~ citations and citation-to-publication (C:P) ~~citation/publication (C/P)~~ ratio. **Figure 3A** shows ~~showed~~ a network map of author citation analysis, with node size proportional to author ~~author's~~ citation counts. Specifically ~~Among them~~, Sanjana Neville E. ranked fourth in citation number ~~citations~~ (n = 4760 ~~4,704 times~~) with relatively few articles (n = 10 ~~12 papers~~). Although the number of articles published by the author was relatively small, the high number of citations indicated that the ~~author’s~~ articles were of high quality and widely recognized by the profession. As shown in ~~you can see from~~ **Table 1**, ~~there was not much difference in the number of articles written by the top 10 most prolific authors. Among them,~~ Li Wei (n = 36 ~~papers~~) ranked first among the top 10 active authors, followed by Hart Traver (n = 22) ~~Zhang Wei (26 papers)~~ and Doench, John G. (n = 21 ~~24 papers~~), who was also the second most cited author (n = 5228).

We also determined ~~found~~ that Zhang Feng was the most cited author (n = 9334 ~~9,188 times~~). Eighteen publications by him had a C:P ratio of 518.56, which ranked first. ~~He had published 19 publications with a C/P ratio of 483.58, also ranking first.~~ In addition, according to our search, Zhang Feng was the corresponding author of the two ~~three~~ most cited papers. The most cited paper ~~of these~~ (n = 3045 ~~2,996 times~~) was an article published by Shalem, Ophir ~~as first author~~ in *Science* in 2014 entitled “Genome-Scale CRISPR-Cas9 Knockout Screening in Human Cells”. Shalem et al. ~~They~~ identified the possibility of negative/positive selection screening in human cells through lentivirus delivery of a genome scale CRISPR-Cas9 knockout library [19]. This work provided a more effective and promising method for targeted screening.

Of note, **Table 1** also shows that ~~showed something quite different:~~ Li Wei, the most productive author, had a C:P ~~C/P~~ ratio of 82.22 ~~78.50~~, which was lower than some authors. Li Li had the lowest citation number ~~times~~ (n = 353 ~~340 times~~) and C:P ~~C/P~~ ratio (n = 18.58 ~~17.00 times~~) among the most productive authors.

**3.2.2 Collaboration of authors**

**Figure 3B** displays ~~displayed~~ a network visualization map of the co-authorship analysis of 91 ~~105~~ authors with at least 9 published articles. Among the cancer studies that utilized CRISPR, ~~We can see that in the field of CRISPR and cancer~~, there were individual researchers in addition to groups of a few academics or many researchers.

~~In addition to the left side,~~ **Figure 3B** depicts numerous ~~was surrounded by many~~ individual researchers ~~scholars~~. Among the researchers ~~them~~, Li Feng and Li Yan were ~~was~~ the most productive individual authors ~~author~~ with 12 publications each, which was more than all other individual researchers and even some small collaborative groups. The most influential individual researcher ~~scholar~~ was Marson Alexander, who had published 10 papers with 1411 ~~1,359~~ citations. ~~Two small research groups were located on the left of the image. One group consisted of Ramakrishna Suresh and Kim Kye-Seong. Another group was composed of Wei Lai and Wong Chun-Ming.~~ Another individual researcher with a high citation number was Wei Lai (n = 1046), who ~~They~~ discovered a key driver of sorafenib ~~Sorafenib~~ resistance in hepatocellular carcinoma treatment through genome-wide CRISPR-Cas9 library screening [20]. The study also indicated that resistance is ~~could be~~ effectively overcome by targeting the key driver.

The center of the illustration displays ~~displayed~~ several large research groups containing most of the researchers ~~scholars~~ and their collaborations. ~~Different colors represented different groups of collaborators, and the connecting lines indicated the close cooperation between them.~~ Most of the ~~these~~ collaborators groups had primary authors, such as Zhang Feng in the yellow ~~bright blue~~ cluster, Li Wei in the brown cluster, ~~orange~~ ~~and~~ Doench John G.~~, Root David E.~~ in the orange cluster and Hart Traver in the blue cluster ~~yellow~~. By comparison, the orange ~~bright blue~~ cluster had the highest total link strength and ~~contained the most top authors. Their~~ the co-authors had published numerous ~~a number of~~ influential articles. For example, these researchers ~~Vazquez Francisca et al.~~ discovered that targeting genomic-amplified regions in cancer cell lines using CRISPR-Cas9 technology ~~may~~ induce DNA damage and G2 cell cycle arrest [21]. This gene-independent anti-proliferative cell response may allow sequence-specific therapeutic strategies to be used in cancer therapy.

The overlay visualization map is ~~was~~ shown in **Figure 3C**. The research group, consisting ~~composed~~ of Chen Sidi and Zhang Feng, started relevant research earlier. One group that has been active lately includes ~~was that of~~ Kim Kye-seong and Ramakrishna Suresh,who ~~. They~~ used CRISPR-Cas9 to conduct ~~carry out~~ genome-scale knockout of the entire ~~whole~~ set of genes encoding ubiquitin-specific protease (USP) and screened USP3 as a deubiquitination enzyme (DUB) for the cell division cycle 25 oncoprotein [22]. This finding may help to screen target proteins of functional DUBs at the genomic scale. In addition to the above collaborator ~~collaborators~~ group, Hou Changjun has maintained a keen interest in CRISPR and cancer as an individual researcher in recent years.

**3.3 Journals and institutions analysis**

**3.3.1 Leading journals**

In total, 645 ~~755~~ journals contributed 4408 cancer-related ~~4,814~~ articles involving ~~on~~ CRISPR ~~in the field of cancer~~. The top 10 journals with the highest output and most citations are ~~were~~ shown in **Figure 4A and 4B**, respectively.

As shown in **Figure 4A**, 2 ~~two~~ journals published > ~~more than~~ 130 ~~150~~ papers, while the remaining journals had a smaller difference in the number of articles published. Among the journals ~~them~~, *Nature Communications* ~~Scientific Reports~~ published the most articles (n = 147 ~~156 papers,~~ [3.34%] ~~3.24%~~) with ~~an IF/JCR quartile of 4.997/Q2. Nature Communications published 153 papers (3.18%) with~~ the highest IF ~~of~~ (17.694), and ranked 4^th^ ~~fourth~~ in citation counts. Among the top 10 journals, *Cancer Death & Disease* had the fewest number of articles (n = 68 ~~70 papers~~) with an ~~the~~ IF/JCR quartile of 9.696/Q1 in 2021. As shown in **Figure 4B**, ~~in terms of citations,~~ *Nature* had the most citations (n = 12,111 ~~11,723 times~~), far surpassing the other top journals. *Science* had the greatest average citation number (351.95), followed by *Nature* (237.63). ~~Nature Communications had the highest H-index of 47, followed by Nature of 41.~~ Among the ~~these~~ 10 journals, one-half of the journals ~~them~~ were published in the UK and the other half were published in the US.

As can be seen from the network visualization of the total link strength of 35 journals (**Figure 4C**), *Nature* and *Nature Communications* remained the leading journals ~~among them~~. In addition, *Genome Biology*, *Cell Reports*, and *Cancer Discovery* also had high total link strengths, with IF/JCR quartiles of 18.010/Q1, 9.995/Q1 and 38.272/Q1, respectively. The overlay map (**Figure 4D**) demonstrated some emerging journals that were also of interest to researchers, such as *Cancers*, *Frontiers in Oncology*, and *International Journal of Molecular Sciences*. In conclusion, researchers can peruse different leading journals according to their needs.

**3.3.2 Collaboration of institutions**

We selected 44 ~~43~~ affiliates with at least 42 ~~45~~ articles for co-authorship analysis and produced network and overlay visualization maps of the ~~their~~ collaboration (**Figure 5A and 5B**). As shown in **Figure 5A** , ~~institutions were distributed in concentrated areas, but generally dispersed. The~~ the most collaborative research units~~,~~ were all in the blue cluster, including ~~included~~ Harvard Medical School, Dana Farber Cancer Institute, and the Broad Institute of the Massachusetts Institute of Technology and Harvard ~~Broad Inst MIT & Harvard~~. These three institutons ~~They~~ were also among the top 5 ~~five~~ most cited institutions, with > ~~more than~~ 10,000 citations each. Harvard Medical School and Dana Farber Cancer Institute were also the top 2 ~~two~~ institutions for publishing the most papers, with 237 ~~256~~ and 149 ~~150~~, respectively. One of these papers established Cre-dependent Rosa26 Cas9 knockin mice to overcome the Cas9 delivery difficulties, thereby enabling the application of Cas9-mediated genome editing *in vivo* [23]. The study performed genome editing on these mice in lung tissues ~~tissue~~ to study the dynamics of multiple mutations during tumorigenesis. ~~, and the~~ The results demonstrated the potential of Cas9 mice to assist in the rapid screening of pathogenic gene mutations in a variety of biological and pathological processes [23]. Another group of closely collaborating research institutions was in the green cluster, consisting of Chinese institutions and universities, such as the Chinese Academy of Sciences and Shanghai Jiao Tong University.

~~Interestingly, the results of~~ The overlay visualization (**Figure 5B**) ~~reflected~~ shows that ~~the aggregation of the institutional distributions similar to the network. The~~ the most collaborative research teams started the study earlier than Chinese institutions. In the latest advance in the field, Chinese institutions reported an alternative strategy for enabling CRISPR-Cas9 delivery to cells or tissues [24]. The strategy involves ~~involved~~ a non-viral delivery of Cas9 protein and single guide RNA (sgRNA) plasmid: a nanocarrier with gold nanoclusters (GNs) as the core and lipids as the shell. Cas9/sgRNA is ~~would be~~ delivered by modifying GNs with the HIV-1-transactivator of the transcription peptide. The study also demonstrated the ability of this strategy in delivering protein-nucleic acid hybrid agents for gene therapy by designing procedures to treat melanoma [24].

**3.4 Countries analysis**

**3.4.1 Leading countries**

All the cancer publications involving ~~on~~ CRISPR ~~in the field of cancer~~ were distributed in 83 ~~85~~ countries/regions. The United States ranked first with 1999 ~~2,152~~ publications (45.34% ~~44.70%~~ of 4408 papers), followed by People’s Republic of China (n = 1436 ~~1,576 publications,~~ [32.58%] ~~32.74%~~), Germany (n = 391 ~~425 publications,~~ [8.87%] ~~8.83%~~), Japan (n = 288 ~~320 publications,~~ [6.53%] ~~6.65%~~) and England (n = 261 ~~288 publications,~~ [5.92%] ~~6.65%) (~~ **;Table 2**). Among these countries, the United States and the People’s Republic of China published > ~~more than~~ 1,000 ~~1,500~~ articles, which are far more than any other country. The annual trends and total number of articles by assessing the 10 most productive countries are ~~were~~ shown in **Figure 6A**. ~~In 2012, South Korean scientists published the first paper on CRISPR and cancer, the only study at that time.~~ We noted that the United States had been the most active country in this area of research ~~field~~ since 2013, followed by the People’s Republic of China, which surpassed the United States in 2021.~~continued to increase the number of articles published.~~ Although other countries have been expanding ~~their~~ annual research publications in recent years, the United States and the People’s Republic of China have long held the number 1 and 2 ~~No.1 and No.2~~ positions, respectively, with respect to ~~in terms of~~ total volume and number of annual papers.

As shown in **Table 2**, ~~in terms of total citations,~~ the six countries with the most published articles (USA, China, Germany, Japan, England, and Canada) were also the six countries with the greatest number of ~~most~~ citations. The United States had greatest impact on area of research, with a total of 87,757 ~~89,696~~ citations, which was greater ~~more~~ than the other 9 ~~nine~~ countries combined. The finding ~~situation~~ changed with respect to ~~when it comes to~~ the number of citations per article ~~and H-index~~ (**Figure 6B and Table 2**). The United States still ranked first among all countries on the list~~,~~ with 43.90 ~~41.68~~ citations per article ~~and an H-index of 136~~. ~~China ranked second in H-index (72), but its~~ ; however, the average per item (20.50 ~~19.08~~ times) of China was lower than the other ~~that of other~~ selected countries, except South Korea. South Korea had the lowest citation number ~~citations~~ (n = 2257 ~~2,853 times~~) and C:P ~~C/P~~ ratio (13.36 ~~15.18~~) among the most productive countries.

**3.4.2 Collaboration of countries**

We selected 40 ~~41~~ countries/regions with at least 9 publication frequencies for co-authorship analysis. As shown in **Figure 6C**, the United States ranked first with respect to ~~in terms of~~ the quantity of publications, citations, and total link strength, followed by the People’s Republic of China and Germany. Larger nodes and thicker linking lines implied that cooperation among leading countries had ~~played~~ an important role in international exchanges. ~~It was found that~~ Countries in European ~~region~~ cooperated more closely with each other than elsewhere. The Netherlands ~~published~~ only had 162 ~~170~~ publications, but ~~it~~ the Netherlands also cooperated extensively with other countries or regions, and the ~~its~~ total link strength was higher than Canada, Japan, South Korea, and other countries.

**Figure 6D** shows ~~displayed~~ an overlay visualization of co-authorship between countries/regions. ~~We can see that~~ The recently active countries/regions were distributed in the periphery, while the countries studied earlier were clustered in the center of the structure. Articles ~~in~~ from Belgium, the United States, and Japan were published earlier than other countries. In contrast, cancer research involving ~~in the~~ CRISPR ~~and cancer field has been~~ were conducted relatively late in South Africa, Saudi Arabia, and Malaysia. Moreover, the ~~cooperation~~ cooperative relationships between countries have declined in recent years and shifted to countries with weaker research efforts.

**3.5 Co-citation and co-occurrence analysis**

**3.5.1 Co-cited references and journals**

**Co-cited references analysis ~~could~~ not only ~~reveal~~ revealed the change ~~of~~ in cancer research focus ~~in the field~~, but also identified the core references of great significance for scientific decision-making in related fields** [17,25]**. In ~~this~~ the current study~~,~~ we used VOSviewer co-citation analysis to filter out the most co-cited references. In Figure 7A, 91 ~~110~~ references were co-cited at least 50 times. We chose a density visualization map to make the ~~their~~ distribution clearer. Table 3 lists ~~listed~~ the top 10 co-cited references ~~on~~ involving CRISPR ~~in~~ or cancer. ~~Half~~ One-half of ~~them~~ the articles were published in *Science* ~~, which demonstrated the journal emphasized both quantity and quality of publications. Moreover,~~ and the corresponding author of the four articles was ~~were~~ Zhang Feng. ~~, which reflected his outstanding academic and guidance ability.~~**

**The most co-cited reference, which is entitled ”Genome engineering using the CRISPR-Cas9 system,”~~,~~ was published by Ran F. Ann in *Nature Protocols* ~~Nat Protoc~~ in 2013 and had 553 co-citations. This study designed experiments and provided a set of tools to describe how to use Cas9 nuclease or nickase for genome editing in eukaryotic cells through homologous or non-homologous DNA repair pathways** [26]**. The second most co-cited reference was ~~is~~ by Cong et al.** [10]**, which was published in *Science* in 2013~~, which~~ and had 540 co-citations. ~~Similar to the first one, this article had the same core value in this field.~~ This paper revealed that short RNAs ~~could guide~~ guided Cas9 nucleases to precisely cleave endogenous genomic loci in human and mouse cells, and Cas9 ~~could~~ also ~~promote~~ promoted homology-directed repair with minimal mutagenic activity by ~~converting~~ conversion into a nicking enzyme** [10]**. These studies ~~Studies~~ demonstrated that the RNA-guided nuclease technology was programmable, ~~easy to program and~~ laying the foundation for the application of CRISPR-Cas in cancer research. ~~had wide application prospects.~~ Another reference with ~~more than~~ > 400 co-citations was published by Shalem Ophir ~~, O~~ in 2014, which was also the most cited article, indicating the great influence and authority of the research.**

**It was worth noting that the majority ~~most~~ of the top co-cited studies ~~literatures~~ were published after 2012 ~~between 2012 and 2014~~, except the Subramanian~~'s~~ Aravind article that was published in 2005. This article was not related to the field of CRISPR and caner, but ~~it~~ developed a Gene Set Enrichment Analysis tool. ~~It was~~ The tool is an analytical method for analyzing molecular profiling data, thus allowing researchers to focus on gene expression data at the level of gene sets** [27]**. Articles with high co-citations reveal the significance of the work ~~revealed the hotspots and directions in the field, and the number of citations disclosed their significance~~, so the relevant references or journals are ~~were~~ worth reading and studying carefully.**

**As can be seen from the density visualization map of journals (Figure 7B), *Nature*, *Cell*, and *Science* are ~~were~~ the journals with the highest number of co-citations, as well as the journals with the highest number of citations. *The* ~~journal~~ *Proceedings of the National Academy of Sciences of the United States*, which had an IF of 12.779 in 2021, ranking 4^th^ ~~fourth~~ in number ~~terms~~ of co-citations with a relatively low number of publications (n = 80, [1.82%] ~~84 articles, 1.75%~~) and citations (n = 3549 ~~3860~~ times) compared to the top 3 journals ~~three~~. Combined with the previous analysis of leading journals, *Nature* and ~~it’s~~ the *Nature* sub-journals ~~had~~ have made significant contributions to CRISPR in cancer research, while being authoritative.**

**3.5.2 Keywords analysis**

Keyword co-occurrence analysis was a complementary tool that ~~could enrich~~ enriches understanding of relevant research hotpots and trends, and ~~predict~~ predicts future research in the field [16]. We selected the “all keywords” unit of the occurrence analysis to display the network and overlay visualization map. The threshold was set as ~~more than~~ > 50 occurrences ~~49 times of occurrence,~~ and 107 ~~110~~ high-frequency keywords were selected from 15,032 keywords (**Figure 8A and 8B**).

**Figure 8A** showed 4 clusters of the keyword network, as shown below: Cluster 1, “expression of inflammatory molecules in the development and progression of cancer” (red), included expression, beta-catenin, breast~~/prostate~~ cancer, carcinoma, proliferation ~~tumorigenesis~~, progression, and inflammation; Cluster 2, “mechanisms of oxidative stress on cancer development” (green), ~~contained~~ including oxidative stress, pathways, apoptosis, mutations, activation, and inhibitor ~~inhibition~~; Cluster 3, “genome editing with CRISPR in cancer” (blue) ~~consisted~~ , consisting of cancer, CRISPR-Cas9, gene editing, human cells, mouse ~~model~~, and immunotherapy; and Cluster 4, “role of DNA methylation of genes in cancer” (yellow), ~~had~~ with DNA methylation, gene-expression, transcription, identification, and differentiation. ~~Among them,~~ Cluster 1 and 2 were shown to be closely related to the study of cancer occurrence, promotion, and progression. Cluster 3 was relevant to the CRISPR-Cas system ~~in the~~ with respect to basic research and ~~therapy~~ therapeutic applications ~~of cancer~~. Cluster 4 was associated with cancer gene modification. Although the general direction of these clusters was summarized, some key words may require ~~revisiting~~ review of the article to understand ~~their~~ the meaning. Among the high frequency keywords, “expression,” “cancer,” and “activation” ranked as the top three ~~in terms~~ for number of occurrences times and total link strength. Therefore, use of CRISPR in cancer research focused on ~~was mainly aimed in~~ these three directions.

The overlay visualization map (**Figure 8B**) showed that early research ~~in the field aimed at~~ was limited to cancer stem cells and several types of cancer, ~~also focused on the~~ then expanded to advances in basic cancer research and clinical applications of CRISPR-Cas-mediated gene editing technology ~~in cancer~~. Over time, research hotspots had been developed and ~~dispersed into~~ included related fields~~. However, we could see that~~ ; however, new research priorities in this field ~~had~~ have not clearly emerged. The light-yellow color of the keyword ‘crispr’ indicated its later average age, which may be because CRISPR-related research had been ongoing compared to other directions. Based on this map, we inferred that related therapeutic ~~and applied~~ research and optimization protocols, such as immunotherapy and delivery methods, may be the next research trend.

**4. Research hotspots and frontiers**

Based on the co-citation references and co-occurrence keywords analysis, we identified disease modeling, novel target discovery, and cancer immunotherapy innovations ~~innovating~~ as research priorities and frontiers ~~about the~~ for CRISPR ~~in the field of cancer~~.

**4.1 CRISPR-Cas system and genetic scissors**

CRISPR-Cas systems exists widely in a broad range of bacterial species and provides rich functional versatility and efficiency for genome editing in eukaryotic cells [28,29]. This gene editing technique, derived from the bacterial immune system, is widely exploited in the type-II CRISPR-Cas9 system of *Streptococcus pyogenes*. The type-II CRISPR system generally consists of CRISPR RNA (crRNA), trans-activating crRNA (tracrRNA), and Cas9 protein. The overall immune response relies on the specific recognition of the protospacer adjacent motif by crRNA~~:~~ (the tracrRNA duplex and the cleavage of targeted DNA sequences by Cas9 protein) [13,30]. To be used in manipulating genomes, the CRISPR system is reprogrammed by combining crRNA with tracrRNA to form sgRNA~~. sgRNA still~~ , which directs Cas9 endonuclease to perform sequence-specific DNA double-strand breaks (DSBs) in target DNA [9,31]. Subsequently, DSBs can be exploited for genetic engineering purposes through two different repaired pathways [32]: homology-directed repair or more frequently non-homologues end joining~~,~~ ; followed by the introduction of precise modifications or small indels into the target sequence [33–35].

Due to the simplicity and efficiency of gene manipulation and the programmability of sgRNA, the CRISPR-Cas system has become a widely used method for mammalian genome editing [36]. ~~Since~~ Because cancer is a genetic disease caused by cumulative genetic/epigenetic aberrations, the potential of this genome editing tool for basic research and clinical applications is particularly evident in cancer research and therapeutics [37].

**4.2 CRISPR-Cas for cancer modeling**

~~In order~~ To identify driver genes and interrogate gene functions in tumorigenesis, progression, and maintenance, the generation of genetically-defined models is a core approach. ~~Unlike in the past,~~ The CRISPR-Cas genetic engineering systems provides rapid, simple, and accurate disease models for studying the genetic determinants of cancer and validating drug targets in immuno-oncology.

**4.2.1 *In vitro* models**

With the efficiency and capability of CRISPR tools, it is feasible to generate *in vitro* or *in vivo* models of cancer with the characteristics of human disease. In addition to performing ~~pharmacological~~ pharmacologic studies and verifying the role of identified genes in cancer cell lines, another common *in  vitro* model, the three-dimensional organoid, has been genome-edited to study tumor biology [38,39]. A recent study ~~research article~~ used CRISPR-based genome engineering to establish primary human gastric cancer organoid models ~~with mutation of~~ a TP53 mutation and ~~knockout of~~ AT-rich interactive domain 1A (ARID1A) knockout [40]. ARID1A knockout organoids clearly ~~elucidate~~ elucidated the mechanism and role of ARID1A deletion in oncogenic transformation of gastric epithelium in the absence of TP53 [41]. Primary human organoids accurately mimic the *in vivo* biology of native cancer. This finding has important implications for personalized anti-cancer medicine and ~~precise~~ precision-targeted drug screening [41], as well as for the discovery of genetic and epigenetic markers and prognosis based on relevant hallmarks [42].

**4.2.2 *In vivo* models**

In addition to ~~study~~ studying cancer-related events in *in vitro* models, CRISPR enables the rapid creation of complex and precise animal disease models. Among these models, the most commonly used is the *in vivo* KO mouse model. CRISPR technology ~~allows not only~~ facilitates the development of transgenic models using engineered mouse embryonic stem cells [43], but also the introduction of all CRISPR components into tissues to induce and recapitulate carcinogenesis caused by ~~certain~~ mutations in somatic cells [44]. For the latter model construction approach, targeting and delivering CRISPR components directly *in vivo* leads to a more rapid generation of diverse cancer models with complicated cancer genotypes than *ex vivo* manipulation and transplantation of cultured cells [14].

Another animal model was created by surgically-transplanting xenograft from a patient into immunodeficient mice. This patient-derived xenograft (PDX) cancer model may maintain the histologic ~~histological~~ heterogeneity of the patient’s tumor [45]. Researchers induced immune deficiency in Sprague-Dawley rats by knockout of Rag1, Rag2, and Il2rg, and established a PDX model of squamous lung cancer using this novel rat model. The grafts recapitulated the histopathological characteristics of the primary tumor in several passes [46]. Overall, CRISPR animal cancer models have played a key role in revealing the basics of tumor initiation, maintenance, and progression. In addition, ~~they~~ CRISPR animal cancer models have become faithful models for testing various anti-cancer agents, as well as mechanisms of detecting drug resistance using CRISPR screening [47,48].

**4.3 CRISPR-Cas for target screening**

With the help of ~~Because~~ improved sgRNA libraries ~~are easy to design and clone~~, CRISPR-Cas knockout screening technology ~~facilitate~~ facilitates the interrogation of cancer-related gene function in various cancer models to discover new therapeutic targets [49,50].

**4.3.1 *In vitro* screening**

Both genome-wide and focused loss-of-function CRISPR screening have been successfully adapted to facilitate the identification of genotype-specific vulnerabilities in cancer cell lines. Several high-throughput CRISPR genetic screening studies of genome-scale lentiviral sgRNA libraries have been established in a variety of cell types [51,52]. In these screening studies, cultured cells can be transfected and knocked out with various types of CRISPR libraries (Cas9+sgRNA) and incubated *in vitro* under the desired experimental conditions [50,51]. Subsequent selection assays resulted in the enrichment or depletion of sgRNAs in the library~~,~~ depending on the targeting genes of candidate tumor suppressor genes [53] or drug sensitivity genes [54], respectively. Next-generation sgRNA sequencing analysis will identify and evaluate "hit" events to recover known targets or discover unknown targets for validation [55]. In addition to genome-wide libraries, the researchers have also developed specific sgRNA libraries. These libraries are able to target kinases/proteins involved in genetic regulation and mediate CRISPR–Cas9–based epigenomic regulatory element screening to improve high-throughput screening of regulatory element activity at the native genomic scale [56].

Advanced screening studies have been conducted with both types of libraries to ~~discover~~ identify genes implicated in sensitivity to therapeutic agents, such as a BRAF inhibitor (vemurafenib) [19] and a nucleotide analogue (6-thioguanin) [50], as well as to reveal novel candidate genes involved in drug resistance ~~with~~ using the drug perturbation method [57]. The knock-in screening has also been applied to identify cancer predisposition mechanisms and potential therapeutic targets. ~~For example~~ Specifically, the gain of ~~function of~~ WW domain function containing protein 1 as a cancer susceptibility gene triggers ~~gene of~~ phosphate and tension homology ~~deleted~~ gene deletion on chromosome ~~ten~~ 10 ubiquitination and inactivation, as well as phosphoinositide 3-kinase signaling hyperactivation [58].

**4.3.2 *In vivo* screening**

In addition to screening in cultured cell lines, CRISPR-mediated screening studies have been conducted *ex vivo* and *in vivo*, as in the animal model construction described above. ~~In~~ *~~ex vivo~~* ~~screening,~~ The genome-wide libraries ~~was~~ were modified in a cell pool, ~~and~~ then transplanted into recipient mice for *ex vivo* screening. In this way, several studies have isolated tumors formed from modified and transplanted cells. Genetic characteristics were screened to identify the effect of different genetic aberrations on tumor development or treatment response [59–61]. In addition, ~~the~~ *ex vivo* screening was used to identify metastasis regulators in non­small cell lung cancer [62]. Using a similar approach, modified cancer cells were used for xenografts to identify genes that mediate the response to anti-cancer immunotherapy [8].

Unlike *ex vivo* modification, *in vivo* CRISPR screening has been performed by direct introduction of sgRNA mutant libraries into non-transformed tissues through adeno-associated virus or hydrodynamic injection [63]. A representative study delivered a genome-wide AAV sgRNA library to the mouse brain ~~of a mice~~ model that could induce Cas9­expression ~~expressing~~ to identify tumor suppressor genes and reveal a subset of cancer drivers in resultant glioblastomas [52]. ~~Anyway,~~ Genome-scale CRISPR screening ~~can accelerate~~ has accelerated the discovery of novel drug targets in cancer through a range of creative approaches [64].

**4.4 CRISPR for cancer immunotherapy**

Cancer immunotherapy is an emerging ~~field~~ method of cancer therapy that has achieved clinical benefits in a variety of cancers by generating a highly specific and powerful immune responses to attack tumors [65, 66]~~. However,~~ ; however, due to ~~because~~ the variable therapeutic efficiency of this new therapy ~~is variable~~, CRISPR technology has been applied to improve the efficacy and safety for cancer immunotherapy.

CRISPR can be used to inactivate immune checkpoint genes in primary T cells, such as ~~those~~ genes encoding programmed cell death 1 (PD-1) and cytotoxic T lymphocyte antigen 4. Moreover, the discovery of immune checkpoints can be performed through CRISPR-based screening, such as the discovery that deletion of the tyrosine protein phosphatase, PTPN2, in melanoma cells sensitizes mice to PD-1 inhibition [63].

In addition to immune checkpoints, CRISPR technology ~~could~~ has potentially ~~revolutionize~~ revolutionized adoptive T cell therapy (ACT). ACT is an immunotherapy with a robust anti-tumor response that ~~manipulated~~ manipulates T cells *ex vivo* to increase ~~their~~ anti-cancer potency. This manipulation ~~included~~ includes the purification and expansion of tumor-infiltrating lymphocytes, as well as the two main therapies currently under investigation~~:~~ (targeted insertion of chimeric antigen receptors [CARs] ~~(CAR)~~ and engineered T cell receptor [TCRs]) ~~(TCR)~~ [67]. The great potential of CAR-T cells in immunotherapy has been ~~proven~~ confirmed in a variety of clinical trials [68,69], such as the complete response induced by CD-19 targeted CAR-T cells in patients with refractory B cell acute lymphoblastic leukemia [69].

Despite the success of CAR-T cell therapy, some limitations remain, such as the cost and complexity of autologous T-cell manipulation [70], the graft-versus-host disease (GVHD) caused by allogenic T cells [71], and the low efficacy of CAR-T cell therapy in solid tumor treatment [72]. ~~However,~~ Genome-editing platforms, especially CRISPR, have emerged as powerful tools to overcome these limitations and improve the anti-tumor efficacy and safety of CAR-T cell therapy **(Figure 9A)**. The CRISPR system can target CAR to the TCRα constant (TRAC) [73] or delete β_2_-microglobulin (β_2_M) [74] to silence the TCR or HLA-I of allogenic T cells. This effect helped reduce ~~It also prevents~~ the risk of graft reactivity and limit rejection of allogenic T cells, thus paving the way for the use of off-the-shelf CAR-T cells [75]. Additionally, CRISPR-Cas9-mediated PD-1, TRAC, and β_2_M polygenic disruption of CAR-T cells ~~showed~~ resulted in enhanced anti-tumor activity in preclinical models of human glioblastoma ~~glioma~~ [76]. This finding suggests a role for CRISPR-mediated PD-1 disruption in addressing CAR-T cell therapy failure in solid tumors due to immunosuppressive tumor microenvironment and CAR-T cell exhaustion.

~~In the case of~~ TCR-T cell therapy, ~~similar to~~ like CARs, T cells can be modified with defined TCRs in response to specific tumor antigens. Moreover, ~~TCR-T cell therapy has shown greater potential against solid tumors than CAR-T cell therapy, and~~ TCRs can recognize intracellular proteins [77]~~. This TCR-T cells therapy property~~ , which expands the range of tumor antigen recognition for TCR-T cells therapy and allows TCRs to target cancer-mutated genes [77]~~. However,~~ This TCR-T cell therapy property shows potential against solid tumors ; however, the mismatch and competition between endogenous TCRs (eTCRs) and transgenic ~~TCR~~ TCRs (~~tgTCR~~ tgTCRs) limit the frequency of tgTCR expression in edited T cells [78, 79]. To address the resulting off-target effects, CRISPR technology **(Figure 9B)** has been used to improve expression and enhance recognition by knocking out endogenous TCRαβ [80]. ~~In a phase I human clinical trial,~~ The combination of multiple CRISPR-Cas9 editing of TRAC, TCRβ constant, and PDCD1 with introduction of a cancer-specific TCR transgene (NY-ESO-1) improved anti-tumor immune responses and reduced TCR mismatches in a phase I human clinical trial [81]. ~~Besides~~ In addition to developing more effective and safer cancer immunotherapy with TCR-T cell therapy, CRISPR can also ~~interfere~~ interferes with immune checkpoint genes. TCR-T cell therapy can overcome gene suppression and enhance anti-tumor immune responses, and its application in human antigen-specific cytotoxic T lymphocytes can improve the anti-tumor function of PD-1 deleted cells [82]. ~~Collectively, CRISPR-Cas-based immunotherapeutic approaches will have more be more effective and safer for the future applications in translational drugs and precise medicine.~~

Other than investigating the above two prevailing therapies, the potential and applications of other immune cellular therapies are beginning to emerge. Specifically, natural killer (NK) cells are ideal candidates due to their direct and non-antigen-specific killing effect on cancer cells. Pomeroy et al. [83] performed high-efficiency gene editing of primary NK cells using CRISPR-Cas9, and reported the improved impact of NK inhibitory signaling molecules (ADAM17) and PD-1 gene knockout on NK cell-based cancer immunotherapy. The study demonstrated the enhanced antibody-dependent cytotoxicity of CRISPR-edited NK cells and provided a universal approach for generating engineered primary NK cells for cancer immunotherapy [83]. In addition to NK cells, the researchers extended CRISPR to macrophage-based immunotherapy. Wang et al. [84] utilized CRISPR knockout screens and several analyses to identify the E3 ubiquitin ligase, Cop1, as a regulator of macrophage infiltration and a target for improving the efficacy of cancer immunotherapy [84]. Another study demonstrated that knockout of signal regulatory protein-α in macrophages by CRISPR prevents immune escape and enhances phagocytosis of tumor cells [85]. Based on such progressions, we anticipate that CRISPR-Cas technology will have more applications in cancer immunotherapeutics and continue to mature in the coming years.

**5. Strengths and limitations**

In this study we performed a systematic and comprehensive analysis of the global scientific literature on CRISPR ~~in the field of~~ as related to cancer. Compared with traditional literature reviews, the bibliometric analysis and application of VOSviewer software improved ~~the~~ objectivity and comprehensiveness~~. However,~~ ; however, there were some notable limitations in this study. First, our analysis only collected documents from the WoS database, resulting in bibliography omissions. Second, the selected literature only included articles published in English from 2013-2022 ~~2012 to 2022~~, which may cause selection and time bias. Third, the search strategies omitted searches for main text and some articles that contained only one aspect of keywords or no keywords. Finally, some recently published high-quality studies may not receive enough attention because they were cited less frequently than classical papers, which inevitably leads to literature omission.

**6. Conclusion**

This study systematically summarized the global cancer publications ~~of~~ involving CRISPR ~~in cancer~~ and investigated the distribution and collaboration of scientific outputs through bibliometric and visual analysis. The analysis revealed a steady upward trend in the number of publications, with the United States and the People’s Republic of China making substantial contributions to the field. The journal with the most publications was *Nature Communications* ~~Scientific Reports~~, while the journal with the most citations was *Nature*. Li Wei and Zhang Feng were the authors with the most publications and citations, respectively. Collaboration among research constituents should be expanded and strengthened to promote academic progress and fill research gaps in this field. We hoped that countries could provide platforms for exchanges and cooperation between researchers and institutions. CRISPR had been at the center of interest in cancer modeling and target discovery. Immunotherapy using CRISPR-Cas system had provided future research directions and may facilitate precise medicine for cancer patients through genetically-defined models. Collectively, our study combined an analysis of overall CRISPR research and a review of specific CRISPR cancer applications, summarizing and predicting research directions that further accurately guide cancer researchers.

**Abbreviations**

CRISPR: Clustered regularly interspaced short palindromic repeats; Cas: CRISPR associated system; WoS: Web of science; IF: Impact factor; JCR: Journal citation reports; USP: Ubiquitin-specific protease; DUB: Deubiquitinating enzyme; AAV: adeno-associated virus; sgRNA: single guide RNA; GNs: gold nanoclusters; crRNA: CRISPR RNA; tracrRNA: Trans-activating crRNA; DSBs: Double strand breaks; ARID1A: AT-rich interactive domain 1A; PDX: Patient-derived xenograft; PD-1: Programmed cell death 1; ACT: Adoptive T cell therapy; CAR: Chimeric antigen receptors; TCR: T cell receptor; GVHD: Graft-versus-host disease; TRAC: TCRα constant; β2M: β2-microglobulin; eTCRs: Endogenous TCRs; tgTCRs: Transgenic TCRs; natural killer: NK;

**Ethical Approval and Consent to participate**

Not applicable.

**Consent for publication**

Not applicable.

**Availability of supporting data**

Not applicable.

**Competing interests**

The authors declare that they have no competing interests.

**Funding**

This work was supported by National Natural Science Foundation of China (Grant No. 82072835) to K Wang, Shenyang Science and Technology Bureau Plan Projects (Grant No. 20-205-4-076) to K Wang, 345 Talent Project of Shengjing Hospital of China Medical University (Grant No. M0366) to K Wang, and Outstanding Scientific Fund of Shengjing Hospital to K Wang.

**Authors’ contributions**

KW and YW conceived the review; XW and GZ reviewed the information. YW arranged the format of the figures. HL, GZ and ZL wrote the manuscript. KW and YW critically reviewed the manuscript. All authors read and approved the final manuscript.

**Acknowledgements**

Not applicable.

**References**

[1] Garraway LA, Lander ES. Lessons from the Cancer Genome. Cell. 2013;153:17–37.

[2] Sánchez-Rivera FJ, Jacks T. Applications of the CRISPR–Cas9 system in cancer biology. Nat Rev Cancer. 2015;15:387–393.

[3] Sung H, Ferlay J, Siegel RL, et al. Global Cancer Statistics 2020: GLOBOCAN Estimates of Incidence and Mortality Worldwide for 36 Cancers in 185 Countries. CA Cancer J Clin. 2021;71:209–249.

[4] Stupp R, Hegi ME, Mason WP, et al. Effects of radiotherapy with concomitant and adjuvant temozolomide versus radiotherapy alone on survival in glioblastoma in a randomised phase III study: 5-year analysis of the EORTC-NCIC trial. Lancet Oncol. 2009;10:459–466.

[5] Vogelstein B, Papadopoulos N, Velculescu VE, et al. Cancer Genome Landscapes. Science. 2013;339:1546–1558.

[6] Pon JR, Marra MA. Driver and Passenger Mutations in Cancer. Annu Rev Pathol Mech Dis. 2015;10:25–50.

[7] Drost J, van Boxtel R, Blokzijl F, et al. Use of CRISPR-modified human stem cell organoids to study the origin of mutational signatures in cancer. Science. 2017;358:234–238.

[8] Manguso RT, Pope HW, Zimmer MD, et al. In vivo CRISPR screening identifies Ptpn2 as a cancer immunotherapy target. Nature. 2017;547:413–418.

[9] Jinek M, Chylinski K, Fonfara I, et al. A Programmable Dual-RNA–Guided DNA Endonuclease in Adaptive Bacterial Immunity. Science. 2012;337:816–821.

[10] Cong L, Ran FA, Cox D, et al. Multiplex genome engineering using CRISPR/Cas systems. Science. 2013;339:819–823.

[11] Zhang H, Qin C, An C, et al. Application of the CRISPR/Cas9-based gene editing technique in basic research, diagnosis, and therapy of cancer. Mol Cancer. 2021;20:126.

[12] Barrangou R, Fremaux C, Deveau H, et al. CRISPR Provides Acquired Resistance Against Viruses in Prokaryotes. Science. 2007;315:1709–1712.

[13] Garneau JE, Dupuis M-È, Villion M, et al. The CRISPR/Cas bacterial immune system cleaves bacteriophage and plasmid DNA. Nature. 2010;468:67–71.

[14] Katti A, Diaz BJ, Caragine CM, et al. CRISPR in cancer biology and therapy. Nat Rev Cancer. 2022;22:259–279.

[15] Wallin JA. Bibliometric Methods: Pitfalls and Possibilities. Basic Htmlent Glyphamp Asciiamp Clin Pharmacol Htmlent Glyphamp Asciiamp Toxicol. 2005;97:261–275.

[16] Donthu N, Kumar S, Mukherjee D, et al. How to conduct a bibliometric analysis: An overview and guidelines. J Bus Res. 2021;133:285–296.

[17] Chen X, Xie H, Wang FL, et al. A bibliometric analysis of natural language processing in medical research. BMC Med Inform Decis Mak. 2018;18:14.

[18] van Eck NJ, Waltman L. Software survey: VOSviewer, a computer program for bibliometric mapping. Scientometrics. 2010;84:523–538.

[19] Shalem O, Sanjana NE, Hartenian E, et al. Genome-Scale CRISPR-Cas9 Knockout Screening in Human Cells. Science. 2014;343:84–87.

[20] Wei L, Lee D, Law C-T, et al. Genome-wide CRISPR/Cas9 library screening identified PHGDH as a critical driver for Sorafenib resistance in HCC. Nat Commun. 2019;10:4681.

[21] Aguirre AJ, Meyers RM, Weir BA, et al. Genomic copy number dictates a gene-independent cell response to CRISPR-Cas9 targeting. Cancer Discov. 2016;6:914–929.

[22] Das S, Chandrasekaran AP, Suresh B, et al. Genome-scale screening of deubiquitinase subfamily identifies USP3 as a stabilizer of Cdc25A regulating cell cycle in cancer. Cell Death Differ. 2020;27:3004–3020.

[23] Platt RJ, Chen S, Zhou Y, et al. CRISPR-Cas9 knockin mice for genome editing and cancer modeling. Cell. 2014;159:440–455.

[24] Wang P, Zhang L, Xie Y, et al. Genome Editing for Cancer Therapy: Delivery of Cas9 Protein/sgRNA Plasmid via a Gold Nanocluster/Lipid Core-Shell Nanocarrier. Adv Sci Weinh Baden-Wurtt Ger. 2017;4:1700175.

[25] Tang C, Liu D, Fan Y, et al. Visualization and bibliometric analysis of cAMP signaling system research trends and hotspots in cancer. J Cancer. 2021;12:358–370.

[26] Ran FA, Hsu PD, Wright J, et al. Genome engineering using the CRISPR-Cas9 system. Nat Protoc. 2013;8:2281–2308.

[27] Subramanian A, Tamayo P, Mootha VK, et al. Gene set enrichment analysis: a knowledge-based approach for interpreting genome-wide expression profiles. Proc Natl Acad Sci U S A. 2005;102:15545–15550.

[28] Adli M. The CRISPR tool kit for genome editing and beyond. Nat Commun. 2018;9:1911.

[29] Hendriks D, Clevers H, Artegiani B. CRISPR-Cas Tools and Their Application in Genetic Engineering of Human Stem Cells and Organoids. Cell Stem Cell. 2020;27:705–731.

[30] Sternberg SH, Redding S, Jinek M, et al. DNA interrogation by the CRISPR RNA-guided endonuclease Cas9. Nature. 2014;507:62–67.

[31] Gasiunas G, Barrangou R, Horvath P, et al. Cas9–crRNA ribonucleoprotein complex mediates specific DNA cleavage for adaptive immunity in bacteria. Proc Natl Acad Sci [Internet]. 2012 [cited 2022 Nov 22];109. Available from: https://pnas.org/doi/full/10.1073/pnas.1208507109.

[32] Cho SW, Kim S, Kim JM, et al. Targeted genome engineering in human cells with the Cas9 RNA-guided endonuclease. Nat Biotechnol. 2013;31:230–232.

[33] Yeh CD, Richardson CD, Corn JE. Advances in genome editing through control of DNA repair pathways. Nat Cell Biol. 2019;21:1468–1478.

[34] Doudna JA, Charpentier E. The new frontier of genome engineering with CRISPR-Cas9. Science. 2014;346:1258096.

[35] Rouet P, Smih F, Jasin M. Introduction of double-strand breaks into the genome of mouse cells by expression of a rare-cutting endonuclease. Mol Cell Biol. 1994;14:8096–8106.

[36] Mali P, Yang L, Esvelt KM, et al. RNA-Guided Human Genome Engineering via Cas9. Science. 2013;339:823–826.

[37] Chen M, Mao A, Xu M, et al. CRISPR-Cas9 for cancer therapy: Opportunities and challenges. Cancer Lett. 2019;447:48–55.

[38] Lin A, Giuliano CJ, Sayles NM, et al. CRISPR/Cas9 mutagenesis invalidates a putative cancer dependency targeted in on-going clinical trials. eLife. 2017;6:e24179.

[39] Wanzel M, Vischedyk JB, Gittler MP, et al. CRISPR-Cas9–based target validation for p53-reactivating model compounds. Nat Chem Biol. 2016;12:22–28.

[40] Lo Y-H, Kolahi KS, Du Y, et al. A CRISPR/Cas9-Engineered *ARID1A* -Deficient Human Gastric Cancer Organoid Model Reveals Essential and Nonessential Modes of Oncogenic Transformation. Cancer Discov. 2021;11:1562–1581.

[41] Lo Y-H, Karlsson K, Kuo CJ. Applications of organoids for cancer biology and precision medicine. Nat Cancer. 2020;1:761–773.

[42] Hay M, Thomas DW, Craighead JL, et al. Clinical development success rates for investigational drugs. Nat Biotechnol. 2014;32:40–51.

[43] Wang H, Yang H, Shivalila CS, et al. One-Step Generation of Mice Carrying Mutations in Multiple Genes by CRISPR/Cas-Mediated Genome Engineering. Cell. 2013;153:910–918.

[44] Winters IP, Murray CW, Winslow MM. Publisher Correction: Towards quantitative and multiplexed in vivo functional cancer genomics. Nat Rev Genet. 2018;19:801.

[45] Chandrasekaran AP, Karapurkar JK, Chung HY, et al. The role of the CRISPR‐Cas system in cancer drug development: Mechanisms of action and therapy. Biotechnol J. 2022;17:2100468.

[46] He D, Zhang J, Wu W, et al. A novel immunodeficient rat model supports human lung cancer xenografts. FASEB J. 2019;33:140–150.

[47] Chen Z, Cheng K, Walton Z, et al. A murine lung cancer co-clinical trial identifies genetic modifiers of therapeutic response. Nature. 2012;483:613–617.

[48] Sánchez-Rivera FJ, Jacks T. Applications of the CRISPR–Cas9 system in cancer biology. Nat Rev Cancer. 2015;15:387–393.

[49] Sanjana NE, Shalem O, Zhang F. Improved vectors and genome-wide libraries for CRISPR screening. Nat Methods. 2014;11:783–784.

[50] Sanson KR, Hanna RE, Hegde M, et al. Optimized libraries for CRISPR-Cas9 genetic screens with multiple modalities. Nat Commun. 2018;9:5416.

[51] Wang T, Wei JJ, Sabatini DM, et al. Genetic screens in human cells using the CRISPR-Cas9 system. Science. 2014;343:80–84.

[52] Wang T, Birsoy K, Hughes NW, et al. Identification and characterization of essential genes in the human genome. Science. 2015;350:1096–1101.

[53] Chow RD, Guzman CD, Wang G, et al. AAV-mediated direct in vivo CRISPR screen identifies functional suppressors in glioblastoma. Nat Neurosci. 2017;20:1329–1341.

[54] Shi J, Wang E, Milazzo JP, et al. Discovery of cancer drug targets by CRISPR-Cas9 screening of protein domains. Nat Biotechnol. 2015;33:661–667.

[55] Yin H, Xue W, Anderson DG. CRISPR–Cas: a tool for cancer research and therapeutics. Nat Rev Clin Oncol. 2019;16:281–295.

[56] Klann TS, Black JB, Chellappan M, et al. CRISPR–Cas9 epigenome editing enables high-throughput screening for functional regulatory elements in the human genome. Nat Biotechnol. 2017;35:561–568.

[57] Krall EB, Wang B, Munoz DM, et al. KEAP1 loss modulates sensitivity to kinase targeted therapy in lung cancer. eLife. 2017;6:e18970.

[58] Lee Y-R, Yehia L, Kishikawa T, et al. WWP1 Gain-of-Function Inactivation of PTEN in Cancer Predisposition. N Engl J Med. 2020;382:2103–2116.

[59] Katigbak A, Cencic R, Robert F, et al. A CRISPR/Cas9 Functional Screen Identifies Rare Tumor Suppressors. Sci Rep. 2016;6:38968.

[60] Kodama M, Kodama T, Newberg JY, et al. In vivo loss-of-function screens identify KPNB1 as a new druggable oncogene in epithelial ovarian cancer. Proc Natl Acad Sci [Internet]. 2017 [cited 2022 Dec 25];114. Available from: https://pnas.org/doi/full/10.1073/pnas.1705441114.

[61] Braun CJ, Bruno PM, Horlbeck MA, et al. Versatile in vivo regulation of tumor phenotypes by dCas9-mediated transcriptional perturbation. Proc Natl Acad Sci [Internet]. 2016 [cited 2022 Dec 25];113. Available from: https://pnas.org/doi/full/10.1073/pnas.1600582113.

[62] Chen S, Sanjana NE, Zheng K, et al. Genome-wide CRISPR Screen in a Mouse Model of Tumor Growth and Metastasis. Cell. 2015;160:1246–1260.

[63] Weber J, Öllinger R, Friedrich M, et al. CRISPR/Cas9 somatic multiplex-mutagenesis for high-throughput functional cancer genomics in mice. Proc Natl Acad Sci. 2015;112:13982–13987.

[64] Tzelepis K, Koike-Yusa H, De Braekeleer E, et al. A CRISPR Dropout Screen Identifies Genetic Vulnerabilities and Therapeutic Targets in Acute Myeloid Leukemia. Cell Rep. 2016;17:1193–1205.

[65] Khalil DN, Smith EL, Brentjens RJ, et al. The future of cancer treatment: immunomodulation, CARs and combination immunotherapy. Nat Rev Clin Oncol. 2016;13:273–290.

[66] Alok A, Seok K, Wesolow. A Case of Abdominal Pain and Diarrhea Post Immunotherapy: Hypophysitis Associated with Immune Checkpoint Inhibitors. J Transl Int Med. 2022;10:178–180.

[67] Maus MV, Fraietta JA, Levine BL, et al. Adoptive immunotherapy for cancer or viruses. Annu Rev Immunol. 2014;32:189–225.

[68] Brudno JN, Kochenderfer JN. Chimeric antigen receptor T-cell therapies for lymphoma. Nat Rev Clin Oncol. 2018;15:31–46.

[69] D’Aloia MM, Zizzari IG, Sacchetti B, et al. CAR-T cells: the long and winding road to solid tumors. Cell Death Dis. 2018;9:282.

[70] Fix SM, Jazaeri AA, Hwu P. Applications of CRISPR Genome Editing to Advance the Next Generation of Adoptive Cell Therapies for Cancer. Cancer Discov. 2021;11:560–574.

[71] Ghosh A, Smith M, James SE, et al. Donor CD19 CAR T cells exert potent graft-versus-lymphoma activity with diminished graft-versus-host activity. Nat Med. 2017;23:242–249.

[72] Lamers CHJ, Sleijfer S, Vulto AG, et al. Treatment of metastatic renal cell carcinoma with autologous T-lymphocytes genetically retargeted against carbonic anhydrase IX: first clinical experience. J Clin Oncol Off J Am Soc Clin Oncol. 2006;24:e20-22.

[73] Eyquem J, Mansilla-Soto J, Giavridis T, et al. Targeting a CAR to the TRAC locus with CRISPR/Cas9 enhances tumour rejection. Nature. 2017;543:113–117.

[74] Ren J, Liu X, Fang C, et al. Multiplex Genome Editing to Generate Universal CAR T Cells Resistant to PD1 Inhibition. Clin Cancer Res Off J Am Assoc Cancer Res. 2017;23:2255–2266.

[75] Liu X, Zhang Y, Cheng C, et al. CRISPR-Cas9-mediated multiplex gene editing in CAR-T cells. Cell Res. 2017;27:154–157.

[76] Choi BD, Yu X, Castano AP, et al. CRISPR-Cas9 disruption of PD-1 enhances activity of universal EGFRvIII CAR T cells in a preclinical model of human glioblastoma. J Immunother Cancer. 2019;7:304.

[77] Morris EC, Stauss HJ. Optimizing T-cell receptor gene therapy for hematologic malignancies. Blood. 2016;127:3305–3311.

[78] Bendle GM, Linnemann C, Hooijkaas AI, et al. Lethal graft-versus-host disease in mouse models of T cell receptor gene therapy. Nat Med. 2010;16:565–570, 1p following 570.

[79] van Loenen MM, de Boer R, Amir AL, et al. Mixed T cell receptor dimers harbor potentially harmful neoreactivity. Proc Natl Acad Sci U S A. 2010;107:10972–10977.

[80] Morton LT, Reijmers RM, Wouters AK, et al. Simultaneous Deletion of Endogenous TCRαβ for TCR Gene Therapy Creates an Improved and Safe Cellular Therapeutic. Mol Ther J Am Soc Gene Ther. 2020;28:64–74.

[81] Stadtmauer EA, Fraietta JA, Davis MM, et al. CRISPR-engineered T cells in patients with refractory cancer. Science. 2020;367:eaba7365.

[82] Zhang C, Peng Y, Hublitz P, et al. Genetic abrogation of immune checkpoints in antigen-specific cytotoxic T-lymphocyte as a potential alternative to blockade immunotherapy. Sci Rep. 2018;8:5549.

[83] Pomeroy EJ, Hunzeker JT, Kluesner MG, et al. A Genetically Engineered Primary Human Natural Killer Cell Platform for Cancer Immunotherapy. Mol Ther. 2020;28:52–63.

[84] Wang X, Tokheim C, Gu SS, et al. In vivo CRISPR screens identify the E3 ligase Cop1 as a modulator of macrophage infiltration and cancer immunotherapy target. Cell. 2021;184:5357-5374.e22.

[85] Ray M, Lee Y-W, Hardie J, et al. CRISPRed Macrophages for Cell-Based Cancer Immunotherapy. Bioconjug Chem. 2018;29:445–450.

**Figure legends**

**Figure 1. Flow diagram of literature screening related to CRISPR in cancer.**

**Figure 2. Annual trends in publications and citations on CRISPR in cancer from 2013 ~~2012~~ to 2022.**

**Figure 3. Bibliometric analysis of leading authors and co-authorships in the field of CRISPR in cancer.**

**(A)** The citation analysis of leading authors. **(B)** Network visualization map of the leading authors collaboration. **(C)** Overlay visualization map of the leading authors collaboration.

**Figure 4. Bibliometric analysis of leading journals** **on CRISPR in cancer.**

**(A)** The top 10 published journals. **(B)** The top 10 cited journals. **(C)** Network visualization map of the leading journals. **(D)** Overlay visualization map of the leading journals.

**Figure 5. Co-authorship analysis of leading organizations** **on CRISPR in cancer.**

**(A)** Network visualization map of the leading organizations collaboration. **(B)** Overlay visualization map of the leading organizations collaboration.

**Figure 6. The analysis of leading countries/regions and co-authorships on CRISPR in cancer.**

**(A)** Annual and total publication volume for the top 10 countries. **(B)** Total citations, and average citations per article ~~and H-index~~ for the top 10 countries. **(C)** Network visualization map of the leading countries collaboration. **(D)** Overlay visualization map of the leading countries collaboration.

**Figure 7. The bibliometric analysis of the co-citation on CRISPR in cancer**

**(A)** Density visualization map of co-cited references. **(B)** Density visualization map of co-cited journals.

**Figure 8. Co-occurrence analysis of all keywords on CRISPR in cancer.**

**(A)** Network visualization map of co-occurrence of the high frequency keywords. **(B)** Overlay visualization map of co-occurrence of the high frequency keywords.

**Figure 9. CRISPR-Cas in adoptive T cell therapy.**

(**A**) CRISPR-Cas in CAR T-cell therapy: knockdown of TRAC and B2M using CRISPR reduces the risk of GVHD and limits the alloreactivity of allogenic T cells, respectively, thus improving the anti-tumor efficacy and safety of CAR-T cell therapy. The findings contributes to the establishment of off-the-shelf CAR-T cells. (**B**) CRISPR-Cas in TCR T-cell therapy: knockdown of endogenous TCRαβ with CRISPR reduces the binding competition and mismatch between tgTCRs and eTCRs, thus reducing off-target effects and increasing the surface expression of tgTCRs. Created with BioRender.com

**
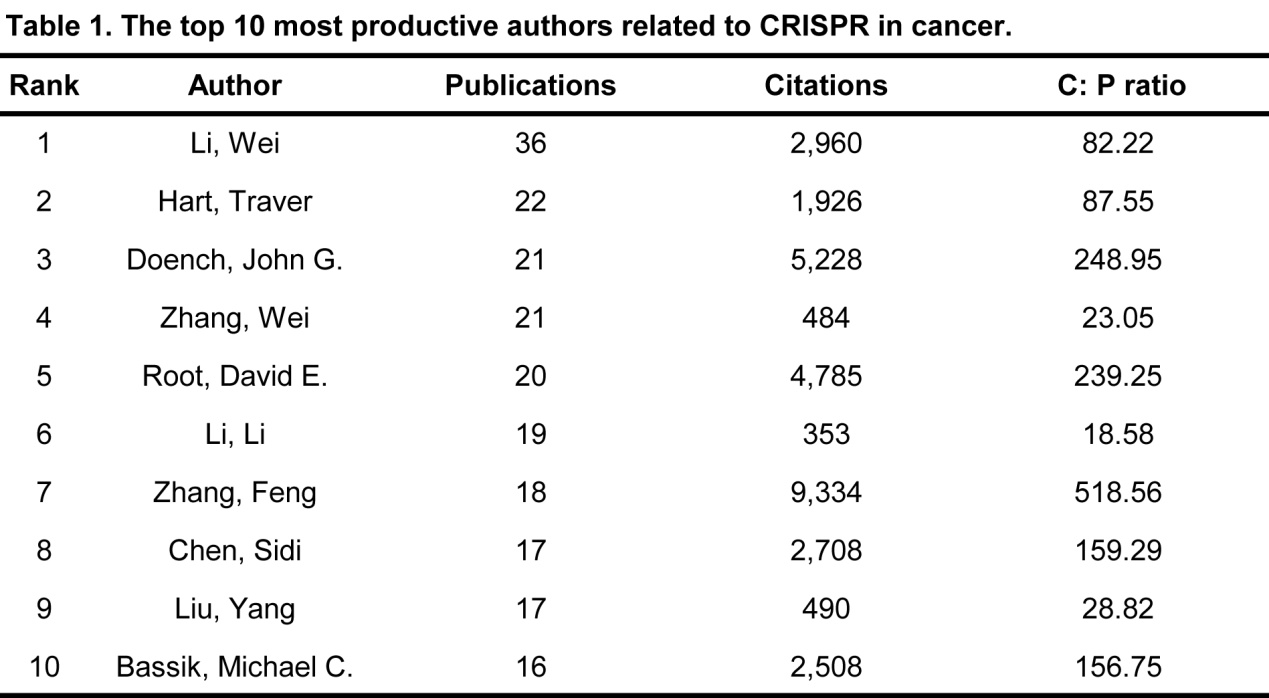
**

**
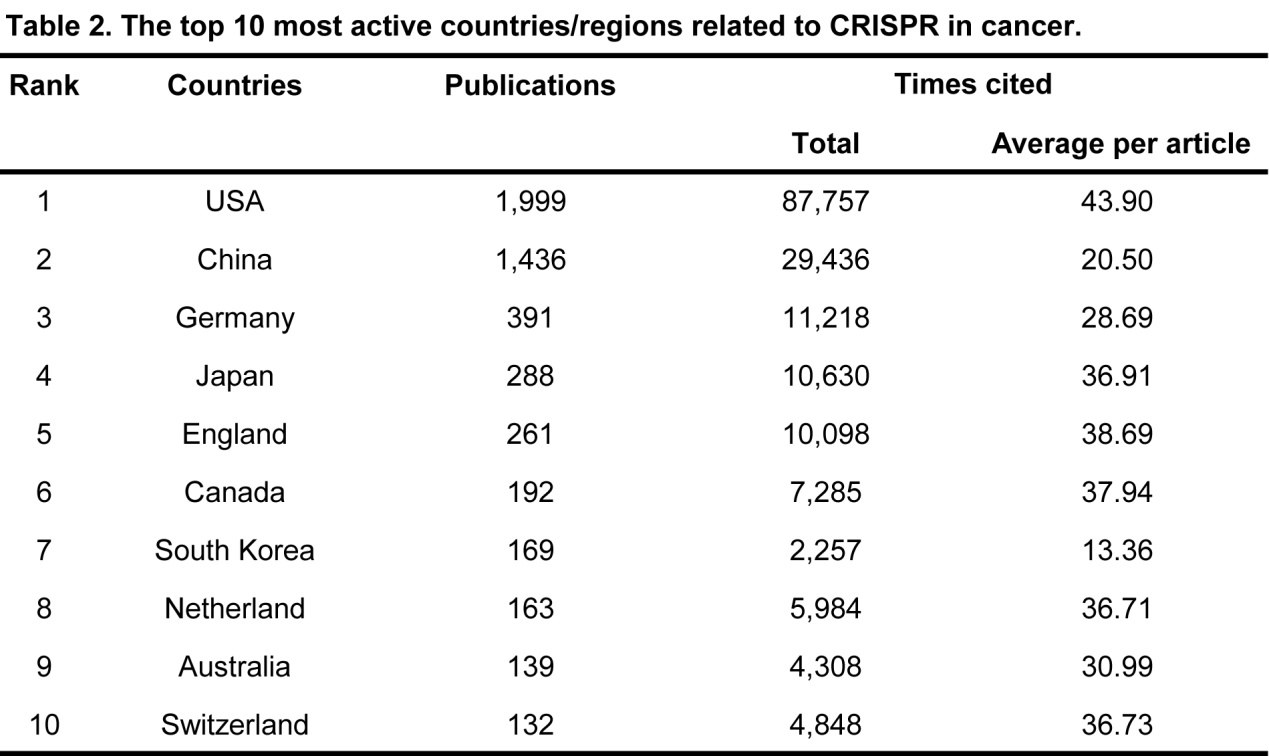
**

**
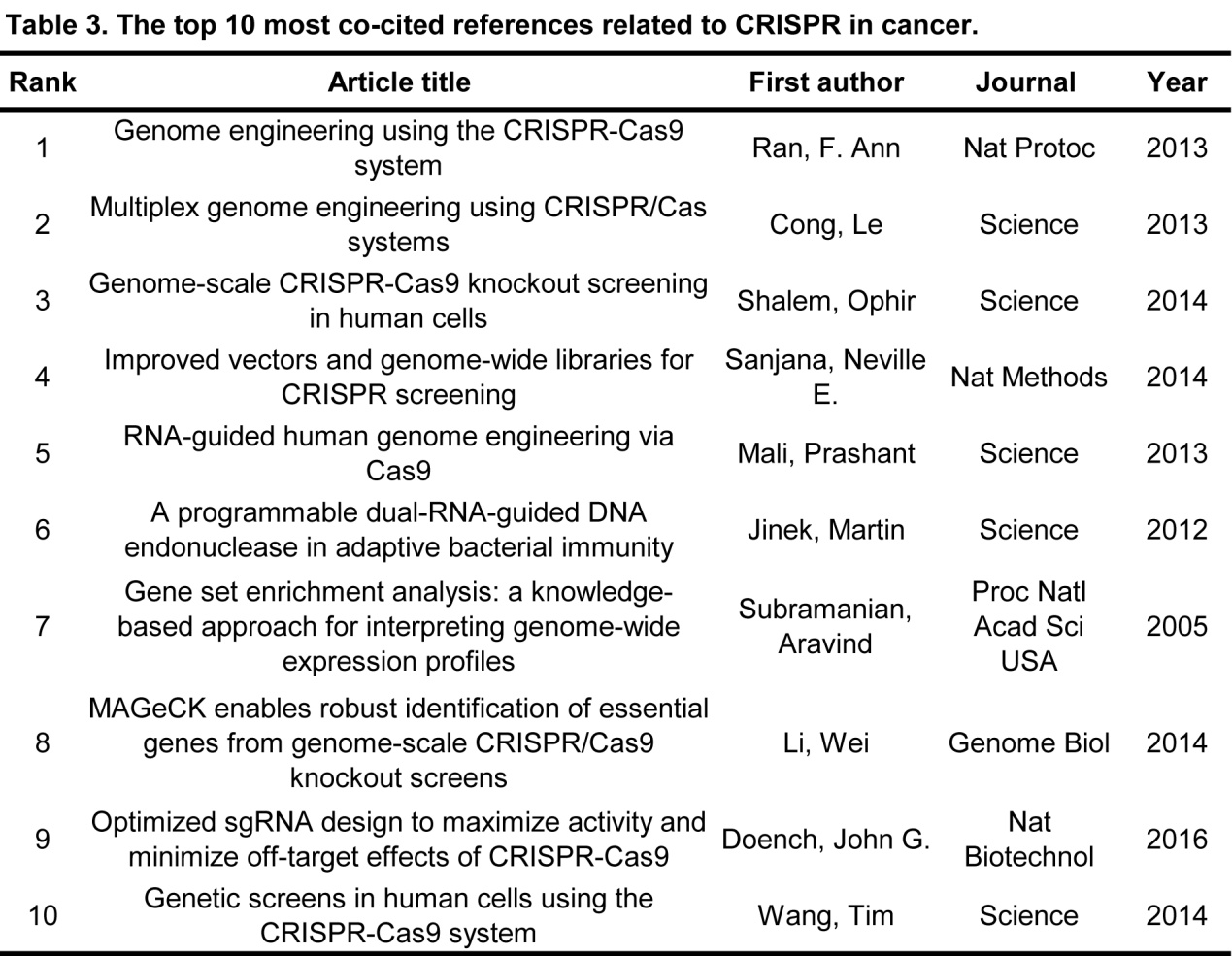
**
